# A high-density integrated map for grapevine based on three mapping populations genotyped by the *Vitis*18K SNP chip

**DOI:** 10.1007/s00122-022-04225-6

**Published:** 2022-10-21

**Authors:** Jessica A. Vervalle, Laura Costantini, Silvia Lorenzi, Massimo Pindo, Riccardo Mora, Giada Bolognesi, Martina Marini, Justin G. Lashbrooke, Ken R. Tobutt, Melané A. Vivier, Rouvay Roodt-Wilding, Maria Stella Grando, Diana Bellin

**Affiliations:** 1 Department of Genetics, Stellenbosch University, Stellenbosch, 7600 South Africa; 2 ARC Infruitec-Nietvoorbij, Stellenbosch, 7599 South Africa; 3 Research and Innovation Centre, Fondazione Edmund Mach, San Michele all’Adige, Italy; 4 Department of Biotechnology, University of Verona, Verona, Italy; 5 South African Grape and Wine Research Institute, Stellenbosch University, Stellenbosch, 7600 South Africa; 6 Center Agriculture Food and Environment (C3A), University of Trento, San Michele all’Adige, Italy

## Abstract

**Key message:**

We present a high-density integrated map for grapevine, allowing refinement and improved understanding of the grapevine genome, while demonstrating the applicability of the *Vitis*18K SNP chip for linkage mapping.

**Abstract:**

The improvement of grapevine through biotechnology requires identification of the molecular bases of target traits by studying marker-trait associations. The *Vitis*18K SNP chip provides a useful genotyping tool for genome-wide marker analysis. Most linkage maps are based on single mapping populations, but an integrated map can increase marker density and show order conservation. Here we present an integrated map based on three mapping populations. The parents consist of the well-known wine cultivars ‘Cabernet Sauvignon’, ‘Corvina’ and ‘Rhine Riesling’, the lesser-known wine variety ‘Deckrot’, and a table grape selection, G1-7720. Three high-density population maps with an average inter-locus gap ranging from 0.74 to 0.99 cM were developed. These maps show high correlations (0.9965–0.9971) with the reference assembly, containing only 93 markers with large order discrepancies compared to expected physical positions, of which a third is consistent across multiple populations. Moreover, the genetic data aid the further refinement of the grapevine genome assembly, by anchoring 104 yet unanchored scaffolds. From these population maps, an integrated map was constructed which includes 6697 molecular markers and reduces the inter-locus gap distance to 0.60 cM, resulting in the densest integrated map for grapevine thus far. A small number of discrepancies, mainly of short distance, involve 88 markers that remain conflictual across maps. The integrated map shows similar collinearity to the reference assembly (0.9974) as the single maps. This high-density map increases our understanding of the grapevine genome and provides a useful tool for its further characterization and the dissection of complex traits.

**Supplementary Information:**

The online version contains supplementary material available at 10.1007/s00122-022-04225-6.

## Introduction

Grapevine, *Vitis vinifera*, is a commercially important fruit crop since the berries are used for a wide range of products such as wine, table grapes, raisins and spirits. Each of these products requires specific berry attributes concerning color, composition and firmness; therefore, grapevine improvement strategies, including breeding programs, have been tailored for such traits. Grapevine cultivation is increasingly prone to the impacts of the changing climate (Duchêne et al. 2010; Hannin 2019), resulting in the introduction of new breeding targets beside more traditional ones such as resistance to biotic stresses (Zyprian et al. 2016). Being a woody perennial fruit crop, grapevine is characterized by a large plant size and long juvenile phase, making it time consuming and costly to grow and evaluate plants for breeding purposes. Therefore, the use of molecular markers in breeding is appealing as seedlings can be selected before reaching maturity, thereby reducing the space, time and money required (Myles 2013).

There are two main strategies to assist breeding with molecular selection: to use markers tightly linked to genomic regions controlling phenotypic traits (marker-assisted selection) or to exploit all available markers as predictors of breeding value (genomic selection) (Barabaschi et al. 2016). In the first approach, linkage maps are used to identify genes and molecular markers associated with traits of interest. Dense linkage maps increase the probability of polymorphic markers in an important chromosome interval, thereby increasing the probability of identifying a marker-trait association. Initially, the construction of linkage maps in grapevine was constrained by the availability of molecular markers (Lodhi et al. 1995; Dalbó et al. 2000; Doligez et al. 2002; Grando et al. 2003). With the introduction of single-nucleotide polymorphisms (SNPs) and cost-effective strategies for SNP genotyping, the number of markers on linkage maps greatly increased (Troggio et al. 2007; Salmaso et al. 2008; Moreira et al. 2011). Current high-throughput genotyping technologies like the Genotyping-By-Sequencing (GBS) approach based on Next-Generation Sequencing (NGS) have allowed the construction of dense linkage maps with thousands of markers (Teh et al. 2017; Torregrosa et al. 2017; Zhu et al. 2018; Lewter et al. 2019; Tello et al. 2019). As with GBS, genotyping arrays, such as SNP hybridization chips, allow for inexpensive and high-throughput genotyping of markers on a genome-wide scale. The lower error rate, and thus higher reproducibility of SNP chip genotyping compared to GBS genotyping, makes it an attractive alternative (Mardis 2008; Houel et al. 2015). Moreover, GBS genotyping is often constrained by the use of different subsets of markers of varying quality. Genotyping by means of a standardized SNP chip can alleviate this problem allowing a faster data integration across populations. Several SNP arrays have been developed for various important fruit crops, such as apple (Chagné et al. 2012; Bianco et al. 2014, 2016), peach (Verde et al. 2012), pear (Montanari et al. 2013, 2019; Li et al. 2019), sweet and sour cherry (Peace et al. 2012) and strawberry (Bassil et al. 2015). In grapevine, following the release of the genome sequence and its annotation and later refinements (The French–Italian Public Consortium for Grapevine Genome Characterization 2007; Canaguier et al. 2017), a first SNP array, the *Vitis*9K SNP chip, was developed based on SNPs identified from resequencing of 11 *Vitis vinifera* cultivars and six wild *Vitis* species (Myles et al. 2010). Subsequently, the GrapeReSeq Consortium developed a second array, the *Vitis*18K SNP array, containing 13561 SNPs identified in 47 *Vitis vinifera* cultivars and 4510 SNPs identified in 12 genotypes from five *Vitis* species and five *Muscadinia rotundifolia* genotypes (Le Paslier et al. 2013). The *Vitis*18K SNP chip has been mostly used to assess population genetic diversity, structure, and relatedness (De Lorenzis et al. 2015, 2019; Degu et al. 2015; Mercati et al. 2016, 2021; Ruffa et al. 2016; Laucou et al. 2018; Marrano et al. 2018; Sunseri et al. 2018; Bianchi et al. 2020; Boccacci et al. 2020; Crespan et al. 2020, 2021; Raimondi et al. 2020; D’Onofrio et al. 2021), or to identify somatic variants to explain intra-varietal diversity (De Lorenzis et al. 2017). The application of the *Vitis*18K SNP array to identify marker-trait associations, however, remains limited (Houel et al. 2015; Duchêne et al. 2020; Mamani et al. 2021). Finally, as for many other species, despite the generation of the high-throughput genotyping tools, the position of genotyped SNPs that can be inferred by sequence information still needs mapping validation.

Published linkage maps are usually based on single mapping populations. This limits the genetic background, and thus the level of polymorphism observed, thereby restricting the number of markers that can be mapped. This can be addressed by the construction of integrated maps, which combine the information from several maps and mapping populations. By identifying markers polymorphic across various genetic backgrounds, integrated maps increase the number of markers on the linkage maps. Furthermore, they enable development of highly validated marker orders across the genomes and are useful in overcoming local loss of genetic resolution by increasing recombination. Reliable information on markers and their ordering supports the identification of chromosomal rearrangements or gene duplications (Maccaferri et al. 2015; Wen et al. 2017). Also, the reliability of marker location provided by mapping and development of reference integrated maps can improve the efficiency of Quantitative Trait Loci (QTL), meta-QTL, genome-wide association (GWAS) studies, gene mapping and cloning, thereby facilitating molecular breeding and selection. To date, some integrated maps have been established in grapevine. Doligez et al. (2006) established the first integrated map containing SSR (simple sequence repeat) markers based on five mapping populations generated from six wine grape parents (‘Bianca’, ‘Cabernet Sauvignon’, ‘Chardonnay’, ‘Grenache’, ‘Riesling’ and ‘Syrah’), and two table grape parents (MTP2121-30 and MTP2223-27). Vezzulli et al. (2008) produced the first grapevine integrated map including SNP markers and AFLPs (amplified fragment length polymorphisms) together with SSRs, by using three mapping populations derived from five wine grape parents (‘Cabernet Sauvignon’, ‘Grenache’, ‘Pinot Noir’, ‘Riesling’ and ‘Syrah’). Subsequently, two integrated maps derived from interspecific hybrids were established. The first, by Moreira et al. (2011), was based on two mapping populations and contained SSR and SNP markers. The marker genotyping for the second map was obtained through GBS and generated an integrated map with only SNP markers based on two half-sib mapping progenies (Teh et al. 2017). The latest grapevine integrated maps were established using GBS-SNP or rhAmpSeq SNP markers. The map by Lewter et al. (2019) used two half-sib muscadine grape mapping populations from the parents ‘Black Beauty’, ‘Nesbitt’ and ‘Supreme’. The map by Tello et al. (2019) used ten half-diallel populations derived from five elite grapevine cultivars. Finally, the map by Zou et al. (2020) was based on four bi-parental populations spanning the diversity of the *Vitis* genus and used markers that were designed by an innovative approach aiming to increase marker transferability across grapevine species.

This study aims to generate the first integrated map using the *Vitis*18K SNP chip based on three mapping populations. The parents of these mapping populations include the well-known wine varieties, ‘Cabernet Sauvignon’ and ‘Rhine Riesling’, the local variety ‘Corvina’, the lesser-known ‘Deckrot’ and a muscat table grape selection (G1-7720). In addition to demonstrating the application of the *Vitis*18K SNP chip across various mapping populations, we confirmed by mapping, the positions for about 9500 of the included SNPs. By constructing a high-density integrated map a reliable order, validated in many cases across multiple maps, was released, while also providing a list of markers with less reliable positioning. This high-density integrated map represents a highly valuable tool that can now be exploited for QTL mapping of relevant traits segregating in the populations.

## Materials and methods

### Mapping populations

Three *Vitis vinifera* mapping populations were used in this study to build the single population genetic maps from which the high-density integrated grapevine map was constructed. The **CSxC** mapping population was derived from a cross made in 2015 by the University of Verona between the ‘Cabernet Sauvignon’ (VIVC 1929) and ‘Corvina Veronese’ (VIVC 2863) varieties, originally including 680 seedlings planted on their own roots in 2017. A population sub-portion including the 142 progenies here considered for mapping purposes was later propagated and planted in two replicates at Società Agricola Vivai Gozzo in Verona, Italy (45° 25′ 45″ N, 11° 01′ 20″ E) on SO4 rootstock. The **DRxG1** mapping population resulted from a cross made by the Agricultural Research Council (ARC) in 2009 between the wine grape ‘Deckrot’ (VIVC 3482) and a table grape selection, G1-7220, originating from a cross between ‘Black Rose’ (VIVC 1408) and ‘Muscat Seedless’ (VIVC 8251). The DRxG1 population, including 225 plants, was planted in 2011 in a single block at ARC Infruitec-Nietvoorbij in Stellenbosch, South Africa (33° 54′ 47.6″ S, 18° 51′ 54.9″ E). A subset of 137 individuals was used for mapping purposes. The **RRxCS** mapping population was obtained from a cross made in 2005 between ‘Riesling Weiss’ (VIVC 10077) and ‘Cabernet Sauvignon’ (VIVC 1929) and was planted in 2008 in the experimental field “Giaroni” of the Fondazione Edmund Mach (FEM) in San Michele all’Adige, Trento, Italy (46° 11′ 36.7″ N, 11° 08′ 12.9″ E). It included 300 vines maintained with standard viticultural practices among which 139 were here considered for mapping purposes. The DRxG1 and CSxC progenies were tested for trueness-to-parent using five SSR markers (VMC6G1, VMC8A7, VMC8B5, VVMD7 and VrZAG79 for DRxG1 and VVMD25, VVMD28, VVMD5, VVS2 and VrZAG79 for CSxC, respectively), whereas the RRxCS progeny was tested using the nine SSR markers recommended for characterization of regional cultivars by the European Project GrapeGen06 (VVS2, VVMD5, VVMD7, VVMD25, VVMD27, VVMD28, VVMD32, VrZAG62 and VrZAG79) (Maul et al. 2012).

### SNP genotyping

The three mapping populations were genotyped with the Illumina *Vitis*18K SNP chip at FEM (San Michele all’Adige, Italy, Table S1). For each of the populations, one individual was genotyped in duplicate to determine reproducibility of the genotyping. Genomic DNA was extracted from 100 mg fresh young green leaves with the QIAGEN ® DNeasy Plant Mini Kit as per the manufacturer’s instructions. Following DNA quantification, using the NanoDrop™ Spectrophotometer 2000 (Thermo Scientific™), 200 nanograms were used for the genotyping assay. Raw SNP data were visualized and clustered in GenomeStudio v2011.1 (Illumina Inc., San Diego, CA, USA) using a GenCall threshold of 0.20. Subsequently, SNPs were re-clustered using ASSiST v1.01 (Di Guardo et al. 2015), which allows for the recovering of SNPs with poor clustering, such as SNPs containing null alleles or additional polymorphisms (multi-allelic clustering). The following quality thresholds were applied to filter the data: SNPs with more than 5% missing data, individuals with a call rate differing more than 10% from the population mean, individuals displaying more than 0.3% unexpected genotypes and SNP markers displaying more than 5% unexpected genotypes or segregation distortion at *P* < 0.001. ASSiST was also beneficial to recode the genotype dataset into JoinMap® input format based on the segregation of the markers in the population (efxeg, hkxhk, lmxll or nnxnp).

### SSR genotyping

In addition to the SNP markers, SSRs were also genotyped mainly to link downstream QTL analyses results to previous results (Table S2). For SSR genotyping, genomic DNA was independently extracted from 100 mg fresh young green leaves using a CTAB extraction (Doyle and Dickson 1987). The CTAB extraction buffer was supplemented with 2% polyvinylpyrrolidone (PVP) and, only in the case of the DRxG1 population, also 0.04 mg/ml proteinase K. For the DRxG1 population, multiplex PCR reactions were prepared using the QIAGEN® Multiplex PCR kit in reaction volumes of 15 µl containing 1X QIAGEN® multiplex PCR master mix, 50 ng of genomic DNA and 0.2 µM of each primer. PCR amplifications were performed as follows: an initial denaturation step of 15 min at 95 °C; followed by 35 cycles of 30 s at 94 °C, 90 s at T_A_ and 60 s at 72 °C; followed by a final extension step of 30 min at 60 °C. SSR genotyping in the CSxC and RRxCS populations was performed as described by Emanuelli et al. (2013). Sizing analysis of PCR products was performed on an ABI 3730xl DNA sequencer (Applied Biosystems) at the Central Analytical Facilities (CAF, Stellenbosch University) using a Genescan 500 LIZ™ or on an ABI 3130xl Genetic Analyzer (Applied Biosystems) using GeneScan™ 400HD ROX™ as internal size standard either at FEM or the University of Verona. Genemapper®v4.1 (Applied Biosystems) was used to score genotypes visually. The datasets were converted in Excel to the JoinMap® input format (abxcd, efxeg, hkxhk, lmxll or nnxnp).

### Theoretical physical position of markers

SNP marker expected positions, when available, were recovered from the literature (Laucou et al. 2018). Alternatively, the flanking sequences of the SNP markers (https://urgi.versailles.inra.fr/Species/Vitis/GrapeReSeq_Illumina_20K) were used as queries for BLASTN (threshold 1xE^−05^) searches to identify their theoretical position on the ‘PN40024 12X.v2’ reference genome sequence (Canaguier et al. 2017) downloaded from https://urgi.versailles.inra.fr/Species/Vitis/Data-Sequences/Genome-sequences. In the case of multiple hits, the best hit (smaller E-value) was selected. For SSR markers, expected physical positions were recovered as described in Delfino et al. (2019) or downloaded from https://urgi.versailles.inra.fr/jbrowse/ from the GFF3 file. Only when the BLASTN search with primer sequences did not provide any output, was the Sequence-Tagged Site (STS) derived from NCBI (https://www.ncbi.nlm.nih.gov/nuccore) used as a query to recover the SSR physical position. The ‘PN40024 12X.v2’ theoretical physical positions for all markers used in this study are reported in Table S1 (SNPs) and Table S2 (SSRs) alongside the mapping information. Linkage disequilibrium (LD) was estimated in Plink v1.90 between all genotyped SNPs with MAF > 0.05 for each chromosome either within each population or considering individuals from all three populations. Average *r*^2^ in sequential bins of 20 Kbp was plotted against physical positions with R 4.2.1.

### Single population map construction

Genotype data of both SNP and SSR markers were used to construct population maps in JoinMap® v5 (Van Ooijen 2019). The dataset for CSxC was flipped around (to CxCS) in order to allow direct comparison of the ‘Cabernet Sauvignon’ maps from CSxC and RRxCS. In all populations, marker data were filtered in JoinMap® to exclude individuals or markers with more than 5% missing data and markers that showed severe segregation distortion (χ2 ≥ 15). This second segregation distortion filtering step was necessary since microsatellites could not be assessed in ASSiST. All further markers were considered to construct single population maps to validate genetic mapping positions. To construct single population maps for map integration, the datasets were reduced by including only a single representative marker per group of markers with 100% similarity (matching genotypes across all individuals that would map to the same position). Representative loci were either selected using the ‘exclude similar loci’ function in JoinMap® that retains the first locus in each group if no shared markers with other datasets were present or manually reduced in MS Excel to ensure that most shared markers across populations were retained as representatives to support downstream map integration.

Markers were grouped into linkage groups using a LOD score of 4.0 or higher. They were ordered using the maximum likelihood (ML) mapping algorithm, which uses the Haldane mapping function (Haldane 1919). The ML parameters were adapted to allow the algorithm to run longer until convergence and were set as follows: chain length = 3000 for S1, S2 and S3, 10,000 for S4, 15,000 for S5 and 30,000 for T0; stop criterion = 5000 for S1, S2 and S3, 20,000 for S4, 25,000 for S5 and 50,000 for T0. The established map quality was inspected using the –log10P, Nearest Neighbor (NN) Fit and NN Stress values. Markers that were identified as a poor fit either due to creating large distances on the map, or to poor map quality criteria (high –log10P, high NN Fit or high NN Stress values) were removed. As explained in the Joinmap manual, poor fitting loci are expected to have values that are very different to the rest of the dataset. In this study, markers with NN Fit and NN stress values greater than 3.00 or markers with -log10P larger than 0.10 were removed. The mapping analysis was repeated until the map contained no poor fitting markers. Excluded markers were then reintroduced one by one to confirm poor fit. If re-introduction of a marker resulted in poor fit criteria or increased the logE-likelihood (indicator of overall map quality), the marker was removed from further analysis until a good quality map was established. As a last step to improve small distance ordering of the markers, the physical theoretical markers positions of representative markers were considered to improve the ordering by providing them as a fixed order input in JoinMap® and checking for similar or improved logE-likelihood values. Again, poor fitting markers were removed from the fixed order, to allow positioning only according to genetic data, and eventually from the map analysis, until the map contained again no poor fitting markers.

### Integrated map construction

The integrated map was developed from the three population maps based on the reduced datasets, as explained previously, which included only one representative marker per group of identical markers. This was implemented to discard redundant information for integration while maximizing shared markers.

The marker orders of the three population maps were inspected visually for collinearity. For each chromosome, the map positions of each marker were compared against each other in MS Excel to identify marker conflicts between population maps. The conflict orders were forced in the other population(s) to test whether the alternative orders were also acceptable. If the forced order resulted in an acceptable map quality (using the same map quality criteria as described previously, similar length maps and similar log-E likelihood), the conflict across maps was considered as inconclusive, and the shared acceptable order was accepted for building the integrated map. Although this sometimes introduced conflicts to the assembly in the other populations, we gave priority to the marker order obtained by the genetic data. Alternatively, if the forced order resulted in poorer map quality or higher stress of markers, the conflict order was not accepted in the other population, resulting in a conclusive conflict between populations. In this case both orders were retained in the respective population for building the integrated map (Maccaferri et al. 2015). The markers involved in these conflicts were differently labeled with suffixes to be included independently in alternative putative positions on the integrated map. The suffixes indicated the conflictual populations, where the population CSxC, DRxG1 or RRxCS was indicated with the letters A, B and C, respectively. This would mean that the suffix –AB was used for conflicts between CSxC and DRxG1, –AC for conflicts between CSxC and RRxCS, and –BC for conflicts between DRxG1 and RRxCS. Prior to map integration, the marker order of each population map was also compared against the ‘PN40024 12X.v2’ reference genome assembly. To also highlight markers and regions that were non-collinear to the assembly, these markers were labeled, for building the integrated map, indicating the population map that was non-collinear to the assembly using the suffixes –A, –B or –C for the CSxC, DRxG1 and RRxCS population respectively.

The integrated map was finally generated by using the MergeMap software (Wu et al. 2011), a package applying a “graph-method”-based approach for building consensus maps. The integrated map was built for each linkage group separately. A weight of 1.0 was applied to each population map for each linkage group. All maps were drawn with MapChart v2.32 (Voorrips 2002).

### Map evaluation

Genome coverage was estimated by mapping the first and last marker on each chromosome to the ‘PN40024 12X.v2’ assembly and determining the percentage of the physical sequence covered by the mapped markers (Tello et al. 2019).

The constructed single population maps and integrated map were tested for collinearity for each chromosome to the physical map of the grapevine ‘PN40024 12X.v2’ reference assembly (Canaguier et al. 2017). This was done by calculating pairwise Spearman rank correlation coefficients for each linkage group with the function cor as implemented in R version 3.5.0. Furthermore, the collinearity was inspected visually with a dot-plot diagram generated by plotting the genetic position of each marker on the linkage groups against the physical position on the reference genome.

Chromosomal distribution of SNP markers that were differently allocated by mapping compared to the 'PN40024 12X.v2' assembly, either on different chromosomes or in the same chromosome but to a different region (inconsistencies > 10 cM), was visually depicted by means of Circos diagrams (Krzywinski et al. 2009). Circos diagrams were prepared as explained at http://circos.ca/. Marker density across the chromosomes of the integrated map was evaluated by counting the number of SNPs in contiguous windows of 5 cM.

## Results

### SNP and SSR genotyping

The parents and progeny from the three mapping populations were genotyped using the *Vitis*18K SNP chip and by SSRs (Table 1). After SNP visualization and clustering in GenomeStudio, ASSiST was employed for data quality filtering, recovering SNPs with poor clustering and re-coding the SNP dataset to JoinMap format. Datasets including 7461, 6641 and 7397 SNPs, respectively, for the CSxC, DRxG1 and RRxCS populations (41.23%, 36.75% and 40.93% of total tested SNPs) were compiled for linkage analysis. These datasets contained 227 (CSxC), 167 (DRxG1) and 258 (RRxCS) SNPs recovered by ASSiST (Di Guardo et al. 2015). Genotyping data were found to be 99.76%, 99.78% and 99.80% reproducible in the CSxC, DRxG1 and RRxCS datasets, respectively. The extent of LD was evaluated in each single population as well as by considering all genotyped individuals from the three populations (Figure S1). Bins of flanking markers in full LD could be found in each single population as well as considering all individuals, but were strongly reduced in this second panel, promising a local increased resolution for mapping by integration. Detailed information on SNP markers, such as the SNP identity, the position on the assembly either available in the literature or recovered by BLASTN of SNP flanking regions to the reference genome, and the ASSIST filtering results including the segregation type and reason for exclusion for each population, is presented in Table S1.

**Table 1:** Summary of information for SNP and SSR markers in three grapevine mapping populations (CSxC: ‘Cabernet Sauvignon’ × ‘Corvina’, DRxG1: ‘Deckrot’ × G1-7720, RRxCS: ‘Rhine Riesling’ × ‘Cabernet Sauvignon’)

	CSxC	DRxG1	RRxCS
*SNP markers excluded during filtering in ASSIST*	*10,610*	*11,430*	*10,674*
Monomorphism	8594	9733	8958
Failed	1586	1558	1528
Segregation distortion	424	132	179
Null allele	6	7	9
*SNP markers included during filtering in ASSIST*	*7461*	*6641*	*7397*
Robust	7234	6474	7139
Recovered multi-allelic SNP	27	18	51
Recovered null allele SNP	200	149	207
*Additional SSR markers included*	*3*	*91*	*64*
**Total n°** **markers** **included in whole dataset** **(representative dataset)** **for linkage analysis in JoinMap®**	**7464 (3616)**	**6732 (3366)**	**7461 (3600)**
*Filtering in JoinMap®*	*0 (0)*	*4 (7)*	*4 (4)*
Excluded due to segregation distortion	0 (0)	1 (1)	2 (2)
Removal of cross-link markers	0 (0)	2 (2)	2 (2)
Ungrouped markers	0 (0)	1 (4)	0 (0)
*Excluded due to poor fit* *during map construction*	*331 (149)*	*402 (186)*	*282 (137)*
**Total n° markers mapped** **in whole dataset** **(representative dataset)**	**7133 (3467)**	**6326 (3173)**	**7175 (3459)**

The table distinguishes marker information for the dataset containing all markers (whole dataset including similar loci), as well as the representative marker dataset (excluding similar loci) used for the computation analyses. Number of markers in representative dataset are given in brackets

Subtotals are in italics, whereas total number of markers of input datasets and total mapped markers are indicated in bold

One hundred and twenty-four SSR markers were genotyped on the parents of at least one of the three mapping populations. SSR genotyping on progenies was carried out for polymorphic SSRs mainly in two of the three populations, and 3, 92 and 64 SSR markers were finally added to the dataset for linkage analysis in CSxC, DRxG1 and RRxCS populations, respectively. Detailed information on SSR markers including SSR name, position on the assembly either available in the literature or recovered by BLASTN, as well as genotyping information is available in Table S2.

### Linkage maps from individual populations

The SNP and SSR genotyping data were combined (Table S3) to construct three population maps. To develop linkage maps 142, 137 and 139 individuals for CSxC, DRxG1 and RRxCS populations, respectively, were used. Since markers were already filtered for segregation distortion in ASSiST, only 3 SNPs were filtered for distortion during map building. On average across the three mapping populations, 13% of the ASSIsT-recovered SNPs were subsequently removed during map building. Although this is higher than the proportion (2%) of poor fit markers in the rest of the robust dataset, which includes only successfully genotyped SNPs, the recovery of SNPs by ASSiST still resulted in the addition of 152–217 SNP markers in the population maps. Furthermore, most poor fit markers (80%) still originated from the robust dataset. In general, segregation was evenly spread between maternal (lmxll) and paternal (nnxnp) segregation, although the DRxG1 parents had fewer shared alleles (segregation types efxeg and hkxhk) compared to the other parental pairs (Table S4).

For the CSxC population, 7133 polymorphic markers, which were represented by 3467 representative markers with fully matching genotypes of the same segregation kind, finally mapped at 1795 unique positions in 19 linkage groups corresponding to the 19 chromosome pairs of grapevine (Table 2, Table S5). The map had a length of 1259 cM with an average inter-locus distance of 0.74 cM and covered 98.26% of the ‘PN40024 12X.v2’ genome assembly. In two chromosomes, there were gaps larger than 10 cM: chr09 had a gap of 16.54 cM and chr19 had a gap of 10.99 cM.

**Table 2:** Summary statistics of the genetic maps constructed in this study for the three grapevine populations (CSxC: ‘Cabernet Sauvignon’ × ‘Corvina’, DRxG1: ‘Deckrot’ × G1-7720, RRxCS: ‘Rhine Riesling’ × ‘Cabernet Sauvignon’)

	CSxC							DRxG1							RRxCS						
	Markers	Representative markers	Unique positions	Length	Gap distance	Largest gap	Correlation	Markers	Representative markers	Unique positions	Length	Gap distance	Largest gap	Correlation	Markers	Representative markers	Unique positions	Length	Gap distance	Largest gap	Correlation
chr01	345	177	100	71.29	0.71	3.01	0.9993	363	172	105	98.89	0.94	4.58	0.9994	340	165	99	81.01	0.82	3.38	0.9987
chr02	318	161	77	52.42	0.68	5.29	0.9992	357	173	93	71.09	0.76	3.44	0.9974	378	166	79	59.71	0.76	2.70	0.9985
chr03	353	135	76	57.70	0.76	3.66	0.9797	318	146	76	72.78	0.96	3.99	0.9924	272	126	81	67.25	0.83	4.51	0.9927
chr04	396	199	112	76.17	0.68	2.16	0.9953	315	154	88	102.20	1.16	13.01	0.9872	388	193	103	85.37	0.83	7.57	0.9955
chr05	404	213	101	65.58	0.65	3.03	0.9994	277	151	97	94.86	0.98	5.55	0.9993	397	217	109	74.86	0.69	2.63	0.9995
chr06	281	143	83	66.11	0.80	9.26	0.9987	238	148	86	89.52	1.04	9.63	0.9997	339	201	110	78.94	0.72	3.35	0.9995
chr07	703	313	155	59.32	0.58	2.54	0.9991	597	304	146	126.87	0.87	5.20	0.9993	634	287	146	108.07	0.74	5.57	0.9996
chr08	309	172	92	70.78	0.77	3.46	0.9995	299	160	98	88.74	0.91	4.58	0.9995	340	183	103	76.59	0.74	3.52	0.9997
chr09	204	85	41	52.85	1.29	16.54	0.9948	306	126	64	56.14	0.88	4.58	0.9975	318	139	59	52.30	0.89	4.38	0.9979
chr10	490	226	115	72.81	0.63	2.54	0.9992	464	249	123	89.09	0.72	2.62	0.9994	483	214	114	97.34	0.85	3.97	0.9992
chr11	333	160	80	60.69	0.76	4.07	0.9975	159	76	47	77.32	1.65	13.82	0.9908	351	174	88	58.20	0.66	6.30	0.9987
chr12	363	180	88	61.26	0.70	2.94	0.9981	284	139	75	80.36	1.07	4.09	0.9985	333	182	89	64.38	0.72	3.73	0.9983
chr13	379	184	86	72.60	0.84	5.24	0.9903	401	210	116	102.33	0.88	7.77	0.9932	401	184	98	90.51	0.92	4.04	0.9852
chr14	458	203	105	75.30	0.72	2.92	0.9992	408	187	111	104.64	0.94	4.97	0.9995	485	214	98	70.59	0.72	2.23	0.9993
chr15	320	178	100	78.12	0.78	2.66	0.9883	193	80	49	63.05	1.29	6.21	0.9985	300	169	87	59.97	0.69	3.76	0.9867
chr16	354	177	83	53.39	0.64	3.33	0.9993	393	190	75	63.69	0.85	3.59	0.9978	305	148	84	60.15	0.72	3.76	0.9991
chr17	308	162	93	58.54	0.63	2.54	0.9990	293	162	89	70.35	0.79	2.66	0.9966	325	139	74	54.96	0.74	8.47	0.9955
chr18	521	253	134	91.87	0.69	4.34	0.9985	421	217	114	142.01	1.25	24.21	0.9997	448	201	105	106.17	1.01	7.27	0.9989
chr19	294	146	74	62.55	0.85	10.99	0.9985	240	129	73	65.86	0.90	5.28	0.9991	338	157	89	66.87	0.75	3.67	0.9992
**Total**	**7133**	**3467**	**1795**	**1259**	**0.74**	**16.54**	**0.9965**	**6326**	**3173**	**1725**	**1660**	**0.99**	**24.21**	**0.9971**	**7175**	**3459**	**1815**	**1413**	**0.78**	**8.47**	**0.9969**

Representative markers are selected from fully matching genotypes of the same kind in progenies. Length and gaps are expressed in cM. The gap distance refers to the average inter-locus gap distance observed on each chromosome. The correlation value refers to the Spearman correlation value with the ‘PN40024 12X.v2’ reference genome assembly

In total, 6326 markers were mapped on the DRxG1 population map (Table 2, Table S5). Initial linkage analysis could not separate markers into 19 linkage groups, and this was solved by removal of the markers 13_23644810 and 9_7465461 cross-linking chr03 and chr13, and chr09 and chr10, respectively. Four markers were unable to group to a linkage group and were excluded. The map contained 1725 unique positions, mapped at an average of 0.99 cM between markers, and included 3173 representative markers. The map had a total length of 1660 cM and a genome coverage of 98.21%. Gaps larger than 10 cM were observed on chr04 (13.01 cM), chr11 (13.82 cM) and chr18 (24.21 cM).

For the RRxCS population, 7175 markers represented by 3459 representative markers were mapped at 1815 positions (Table 2, Table S5). Removal of markers Un_19084121 and 19_2008085 allowed the separation of chr01 and chr04, and of chr16 and chr19, to give 19 linkage groups corresponding to chromosomes. The map had a length of 1413 cM with an average distance of 0.78 cM between markers. The largest gap was observed on chr17 (8.47 cM). The map displayed a genome coverage of 98.17% compared to the ‘PN40024 12X.v2’ assembly.

**Fig. 1:**
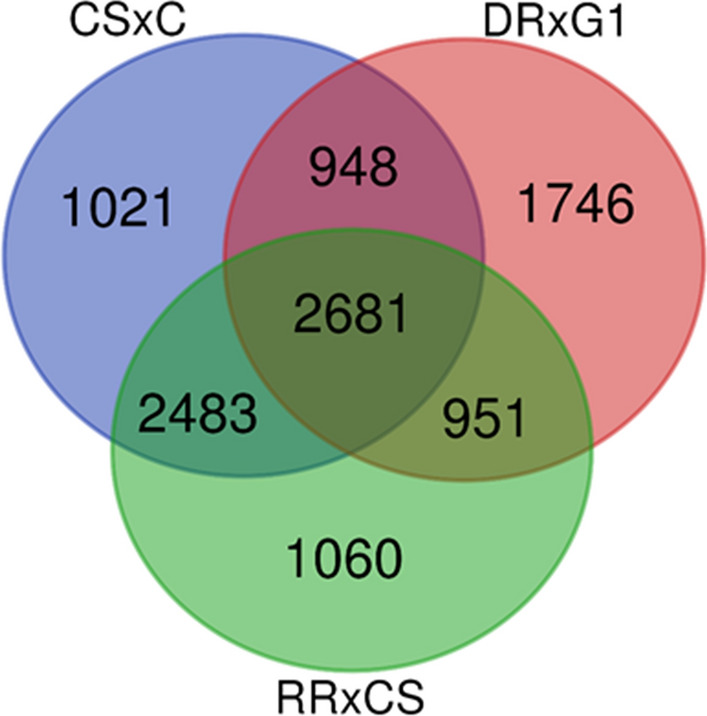
Overview of shared and unique informative markers across the three grapevine mapping populations (CSxC: ‘Cabernet Sauvignon’ × ‘Corvina’, DRxG1: ‘Deckrot’ × G1-7720 and RRxCS: ‘Rhine Riesling’ × ‘Cabernet Sauvignon’) of the 10890 informative markers in this study

Among 10890 informative markers across the three mapping populations, 3827 markers (35.14%) were mapped only in a single population whereas 4382 markers (40.24%) and 2681 markers (24.62%) were shared by two and three maps, respectively. As expected, due to the common parent, the CSxC and RRxCS maps shared more markers (22.80%) than CSxC and DRxG1 (8.71%) or RRxCS and DRxG1 (8.73%). Similarly, population DRxG1 contained more unique markers (16.03%) than CSxC and RRxCS (9.38% and 9.73%) (Fig. 1).

### Comparing single population maps with the reference genome assembly

To evaluate the population map building approach and identify possible discrepancies, the marker order in each map was compared to the order deduced according to their theoretical physical positions in the grapevine reference genome assembly (Canaguier et al. 2017).

Although differences were expected, either due to real genetic divergence of parental genotypes compared to the reference, due to general problems in the assembly or due to inaccurate BLAST-based definition of theoretical positions, the expected position of the SNPs on the reference genome was still considered, in a first instance, to evaluate the mapping approach. The correlation coefficients between the map marker orders and that in the assembly revealed substantial marker order consistencies. Occasionally, slightly lower correlation values were observed in particular chromosomes (see specifically chromosomes 3, 13 and 15 with lower values consistently detected in multiple population maps), but in all chromosomes the correlation was equal or higher than 0.9797, with an average value of at least 0.9965 (Table 2). High correlations were also observed irrespective of whether or not the expected SNP order according to the reference was included to be used by the software in the first spatial sample for running the ML algorithm (see Table S6 for correlations without considering any order information as compared to Table 2 that included this information). The only exception was for the map of chromosome 8 of DRxG1, for which marker ordering was highly divergent compared to the reference when maps were compiled without any SNP order information (Table S6a and b). Because of that, and since including marker order information in general aided the compilation providing slightly shorter and better quality maps (according to NN Fit/NN Stress values), in the previous section we considered as final population maps those built providing the SNP order information as starting point for the algorithm.

**Fig. 2:**
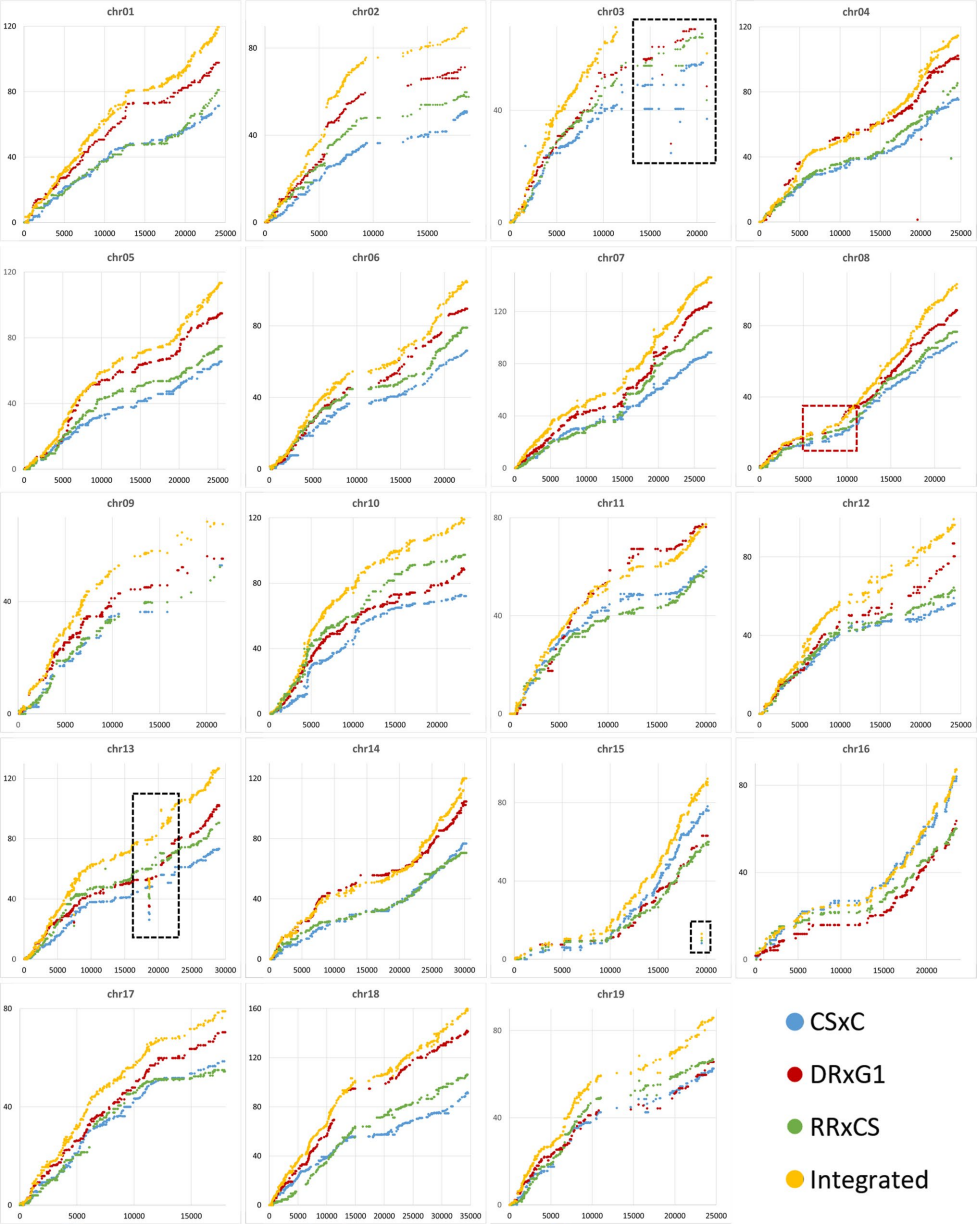
Comparison of the genetic distance on the* y* axis (in cM) against the physical distance of the ‘PN40024 12X.v2’ assembly on the *x* axis (in bp) of the three populations (CSxC (‘Cabernet Sauvignon’ × ‘Corvina’) in blue, DRxG1 (‘Deckrot’ × G1-7720) in red, RRxCS (‘Rhine Riesling’ × ‘Cabernet Sauvignon’) in green) and integrated (in yellow) grapevine maps. Abbreviation: chr = chromosome. The black dashed boxes indicate inconsistencies between genetic and physical distances, while the red dashed block shows the homozygosity stretch on chromosome 8

Even though a substantial marker order consistency to the reference was revealed by correlation coefficients, closer inspection revealed that 2721 (38% of all informative markers), 1994 (32%) and 2731 (38%) markers were involved in conflictual ordering across the 19 chromosomes in the CSxC, DRxG1 and RRxCS maps, respectively. The collinearity between the population maps and reference genome assembly was also further investigated by plotting genetic *vs* physical distances (Fig. 2). These plots also revealed extensive collinearity, despite the reported high number of conflicts. These analyses all indicate that most conflicts likely represent short distance rearrangements. Indeed, only a few signatures of larger distance inconsistencies could be clearly observed in these plots on chromosomes 3, 13 and 15, in agreement with the slightly lower correlation coefficients observed for these chromosomes (Table 2). No signature of inconsistency was observed, as expected, on chromosome 8 for the DRxG1 population map obtained with the integration of the SNP order information from the reference. Additionally, markers showed a characteristic pattern in these plots, with recombination rates varying along the chromosomes and typically reduced around centromeric regions. On most chromosomes, the CSxC and RRxCS maps had comparable collinearity with the physical map, while the DRxG1 map showed greater genetic distances compared to the other two.

**Fig. 3:**
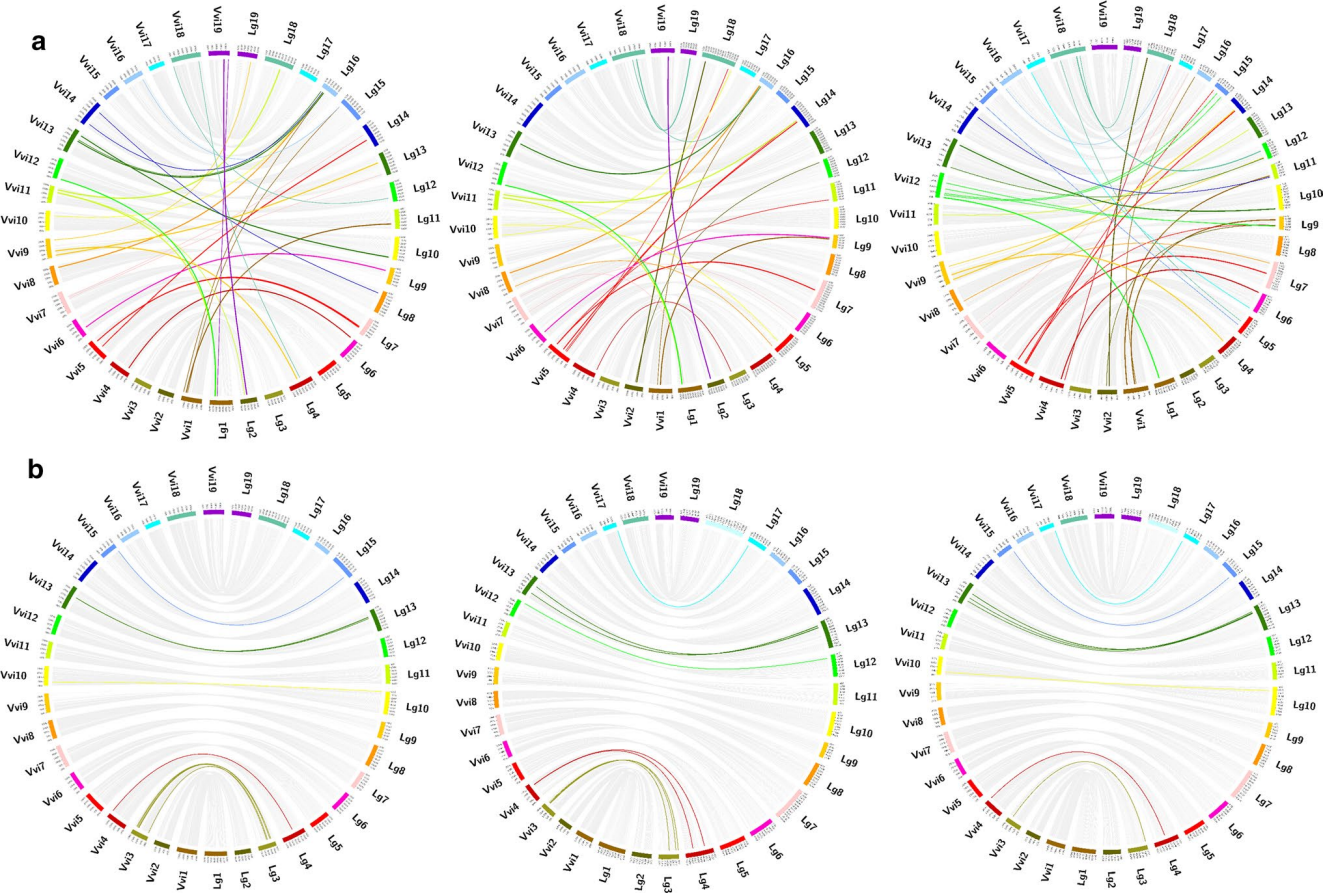
Visualization of markers non-collinear to the 'PN40024 12X.v2' assembly for the three grapevine population maps (CSxC: ‘Cabernet Sauvignon’ × ‘Corvina’, DRxG1: ‘Deckrot’ × G1-7720 and RRxCS: ‘Rhine Riesling’ × ‘Cabernet Sauvignon’): **a** colored ribbons highlight markers that mapped to a different chromosome from that expected according to theoretical chromosome assignment and **b** colored ribbons highlight markers that mapped to the expected chromosome but showed a map position inconsistency greater than 10 cM. ‘PN40024 12X.v2’ chromosomes are designated with the prefix Vvi (left side), whereas the map chromosomes are designated with the prefix Lg (right side). Different colors are provided for non-collinear markers according to the expected chromosome location on the ‘PN40024 12X.v2’ genome assembly. Gray lines indicate collinear markers

The comparison to the reference genome assembly was then used to enquire specific inconsistencies. A detailed investigation identified a group, including only 66 markers in the three different populations (29 in the CSxC population, 27 in the DRxG1 population and 33 in the RRxCS population), which were allocated, on the basis of linkage mapping, to a chromosome different from that of their expected physical position (Fig. 3a, Table S7a). For 16 of these markers, the alternative anchoring was confirmed in more than one population, while 46 markers were mapped in only one population and four SNPs (1_22328219, cn_18_21727890, mu_11_16646972 and 9_2395543) provided inconsistent results in the different populations. The distribution of markers with incorrect theoretical chromosome assignment was in general randomly scattered throughout the chromosomes and genomes without any clear pattern. In only one instance, on chromosome 5, a cluster of two markers that were physically linked (5_20313780 and 5_20326151), relocated to adjacent genetic positions on linkage group 7 of the CSxC and DRxG1 maps.

Focusing on markers that showed large distance order inconsistencies (exceeding 10 cM of distance in allocations inside the expected chromosome) 27 additional markers located on chromosomes 3, 4, 10, 12, 13, 15 and 17 were identified (17 mapped in CSxC population, 18 in DRxG1 population and 16 in RRxCS population (Fig. 3b, Table S8a)). Such discrepancies were still observable despite the marker order information provided during map compilation, and some were related to the signatures of inconsistencies already highlighted in Fig. 2. For 10 of these SNPs, the inconsistency in allocation was confirmed in two populations and for five it was confirmed in all three populations (lb_3_9414759, lb_4_37346, Un_17289290, Un_17310288 and Un_17383522). Investigation of the distribution of the markers displaying inconsistencies of more than 10 cM revealed a cluster of nine associated SNPs (Un_17252166, Un_17274294, Un_17289290, Un_17294697, Un_17303121, Un_17310288, Un_17326323, Un_17337482 and Un_17383522) on chromosome 13. These markers were positioned close to each other in both the physical sequence and on the maps, but at a different position in all maps, hinting at a genomic sequence region which might have been incorrectly located or orientated in the assembly. Moreover, two smaller clusters of associated markers were also identified: again, on chromosome 13 but in a different region (Un_38650425 and Un_38651129), and on chromosome 3 (cn_3_15425566 and 3_14558685).

Finally, 281 SNP markers, physically assigned to the so-called chrUn grapevine chromosome of the ‘PN40024 12X.v2’ genome assembly, were genetically mapped to grapevine chromosomes (Table S9). Most of these markers were mapped to regions of chromosomes 2, 7 and 10. For 193 markers mapping was confirmed in more than one population. Interestingly, by comparison to the ‘PN40024 12X.v2’ genome assembly we found that the genetic mapping was always consistent inside the different scaffolds. In detail, among 104 newly anchored scaffolds, 86 were anchored to the genome in at least two different populations (59 anchored by more markers and 27 by just one marker, respectively). Eighteen scaffolds were anchored in only one of the populations.

### Integrated map

Population maps including only one representative marker per group of identical markers were considered for the purpose of building an integrated map. We considered 6697 representative informative markers, of which 3467, 3173 and 3459 were mapped in the CSxC, DRxG1 and RRxCS populations, respectively. Of these 2023 markers mapped in two populations, and 690 markers mapped in all three populations.

The marker orders of the population maps were compared to identify non-collinear regions to be preliminary addressed to avoid integrated map inflation due to conflicts. Of these non-collinear regions, 65% (51 regions) could be solved since the alternative marker order was of acceptable quality in the other population(s), indicating that marker order between population maps was largely conserved. The remaining 35% (31 regions) resulted in a conclusive conflict, meaning that the alternative order was not acceptable in the other population map(s) (Table S10). This involved 88 markers distributed on 14 of the 19 grapevine chromosomes. The highest number of these conflicts was on chromosomes 7 (13 markers), 4 and 15 (10 markers), 3 (8 markers) and 13 and 10 (7 markers). These non-collinear markers were subsequently treated as independent markers and were mapped in more than one position in the integrated map (indicated with two letter suffix, Table S10, labeled in red).

Despite the high over-all collinearity of the population maps to the ‘PN40024 12X.v2’ genome assembly described previously (Table 2 and Fig. 2), we inspected each population map for conflicts with the assembly to also indicate these in the integrated map. Altogether 1286 markers were involved in generating conflicts in at least one of the population maps, based on the reduced datasets (Table S11). As reported above for the maps based on the whole set of markers, only a few markers were involved in large conflicts (see Table S7 and Table S8 for a comprehensive list of such markers). Instead, we observed that a large majority of these markers were grouped in short conflictual areas, highlighting specific regions for each of the three population maps. Information on these conflictual markers has been retained in the integrated map (labeled with suffixes –A, –B and –C in blue, Table S12). These suffixes provide an indication of the reliability of the integrated map in different regions. Among these conflictual markers for only 40, distributed in 18 regions, the alternative marker order compared to the assembly was consistently supported by more than one map (Table S11, dark blue). This again demonstrates that the majority of conflicts to the assembly consist of short distance rearrangements that are observed in only one population map (Table S11, light blue).

**Fig. 4:**
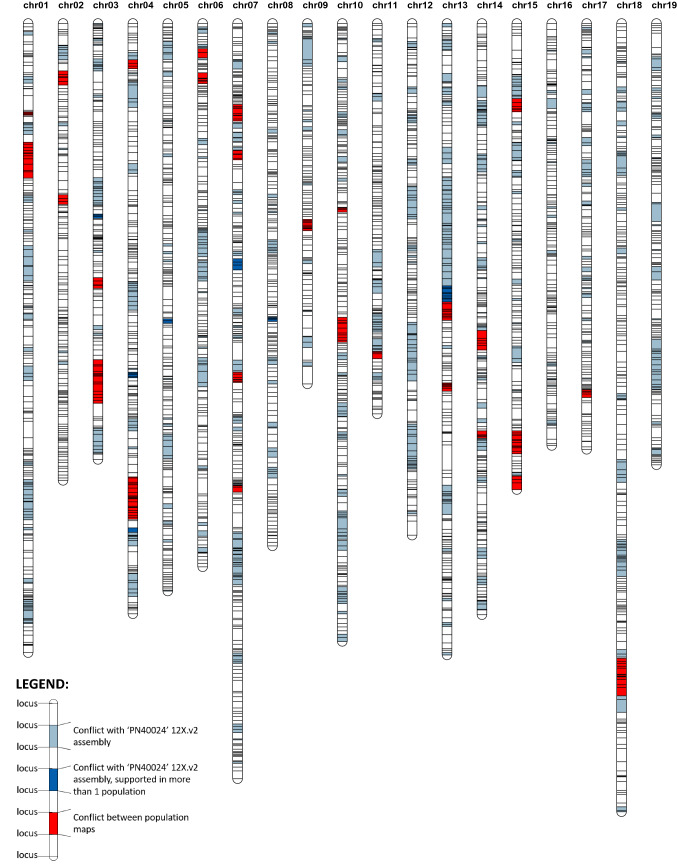
Grapevine integrated map based on three mapping populations (CSxC: ‘Cabernet Sauvignon’ × ‘Corvina’, DRxG1: ‘Deckrot’ × G1-7720 and RRxCS: ‘Rhine Riesling’ × ‘Cabernet Sauvignon’). Marker regions that are in conflict with the ‘PN40024 12X.v2’ assembly are indicated in blue (dark blue if supported by more than one population map, with exception of those introduced by forcing orders during the between maps conflict solving step), whereas marker regions in conflict between population maps are indicated in red. List of these markers are given in Tables S11 and S10 respectively. Marker positions are indicated with horizontal lines. Abbreviation: chr = chromosome

After all different conflict regions were inspected, the final population maps were used to establish an integrated map (Fig. 4, Table S12, Figure S2). The integrated map contained all 6697 representative markers, which mapped at 3492 unique positions on 19 chromosomes, with a final total length of 2094.86 cM and an average inter-locus distance of 0.6 cM. Importantly this integrated map included 2713 anchor markers mapped in at least two maps and 3984 singletons. The largest gap was of 5.12 cM on chromosome 13 and all other gaps were smaller than 4.29 cM (Table 3).

**Table 3:** Summary statistics of the integrated map based on three population maps of grapevine (CSxC: ‘Cabernet Sauvignon’ × ‘Corvina’, DRxG1: ‘Deckrot’ × G1-7720 and RRxCS: ‘Rhine Riesling’ × ‘Cabernet Sauvignon’)

	Total mapped markers	Unique positions	Length	Inter-locus gap distance	Largest gap	Correlation to assembly	Coverage (%)
chr01	358^a^	210	129.69	0.62	3.38	0.9997	99.02
chr02	319	159	94.18	0.59	2.34	0.9996	98.62
chr03	276	161	89.89	0.56	2.81	0.9976	99.34
chr04	362	195	124.25	0.64	3.03	0.9997	99.65
chr05	380	205	116.96	0.57	2.63	0.9998	98.99
chr06	328	180	112.02	0.62	3.36	0.9997	99.34
chr07	579	281	155.71	0.55	3.65	0.9998	99.49
chr08	337	183	107.69	0.59	1.86	0.9998	99.40
chr09	243	117	74.32	0.64	4.29	0.9959	93.84
chr10	458	230	127.41	0.55	2.27	0.9995	98.36
chr11	273	143	80.51	0.56	2.50	0.9993	99.41
chr12	340^b^	165	105.54	0.64	2.94	0.9995	99.73
chr13	376	201	130.24	0.65	5.12	0.9912	99.56
chr14	390^c^	206	121.94	0.59	2.47	0.9997	99.40
chr15	281	156	96.12	0.62	3.02	0.9861	98.76
chr16	344^a^	156	86.97	0.56	2.26	0.9919	99.16
chr17	301	151	87.84	0.58	2.63	0.9994	96.19
chr18	441^c^	229	162.54	0.71	2.66	0.9996	99.64
chr19	314^b^	164	91.04	0.56	3.67	0.9978	99.78
Total	6697	3492	2094.86	0.60	5.12	0.9974	98.82

Length and gaps are expressed in cM

^a^Marker 1_22328219 maped on both chr01 and chr16

^b^Marker cn_18_21727890 maped on both chr12 and chr19

^c^Marker mu_11_16646972 maped on both chr14 and chr18, depending on the population map. These markers were only considered once in the final marker count

**Fig. 5:**
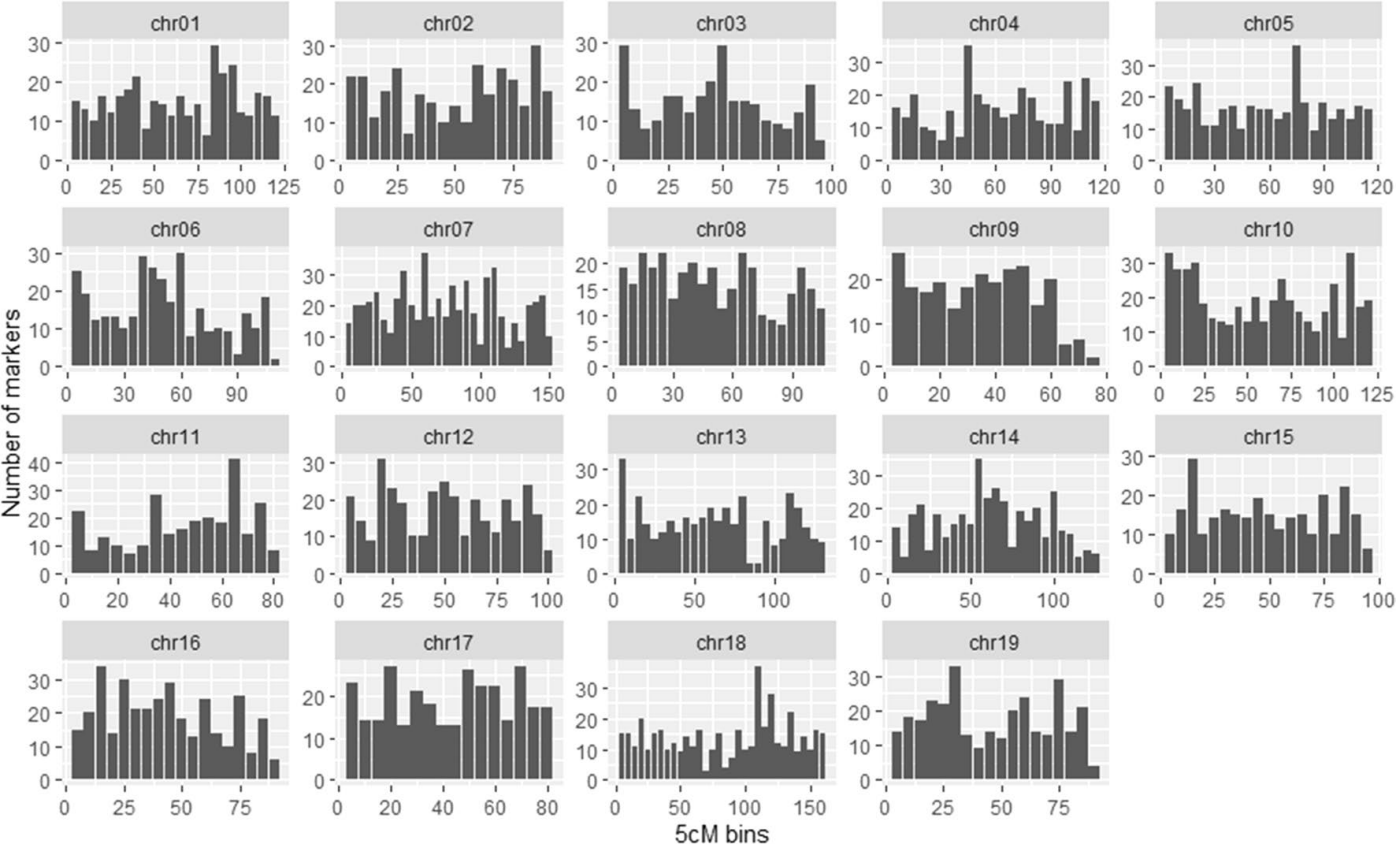
Distribution of marker density on the grapevine integrated map. Marker distribution is reported along the chromosomes (chr) with respect to 5-cM bins

The integrated map displayed an even distribution of markers along each chromosome (Fig. 5). On average, the 5-cM bins contained 16.34 markers per bin. Areas on the map densely populated with more than 35 markers per 5-cM bin could be found on chr05 (75–80 cM), chr07 (60–65 cM), chr11 (65–70 cM), chr14 (55–60 cM) and chr18 (110–115 cM). Areas which were scarcely populated were found mainly on telomeric regions of chr06 (90–95 cM), chr09 (75–80 cM), chr13 (85–95 cM), chr18 (70–75 and 85–90 cM) and chr19 (90–95 cM). The high correlation of the integrated map with the population maps (average correlation coefficient over 0.999904) demonstrates that the integrated map properly reflects the component maps (Table S13).

By considering the physical position of the terminal markers on each chromosome, the integrated map was estimated to cover 98.82% of the genome, which was quite uniform across chromosomes, with the only exception being chromosome 9 (93.84%, Table 3). Similar to the single population maps, the integrated map showed high collinearity with the ‘PN40024 12X.v2’ assembly (Fig. 2). As the integrated map is based on the genetic distances within the population maps, the larger distances observed in the DRxG1 population map were also reflected in the integrated map. Interestingly, the correlations between genetic positions and physical positions were even higher in the integrated map chromosomes compared to the population map chromosomes (Table 3).

## Discussion

### The *Vitis*18K SNP chip as genotyping tool

The *Vitis*18K SNP chip allows for high-throughput genotyping of 18071 SNPs distributed throughout the grapevine genome (Le Paslier et al. 2013). On the technical side, a high successful genotyping rate, i.e., the percentage of retained SNPs after removal of low quality and no-call loci, varying from 91.2% in CSxC to 91.5% in RRxCS (Table 1), was observed. These genotyping rates were close to the upper limit of the range 55.6–92.7% observed in previous studies applying similar thresholds for SNP filtering when using the *Vitis*18K SNP chip (De Lorenzis et al. 2015, 2017, 2019; Mercati et al. 2016; Ruffa et al. 2016; Sunseri et al. 2018; Bianchi et al. 2020). This can probably be attributed to the large set of genotypes used to classify SNPs and the fact that we analyzed full sib progenies. ASSiST considers the pedigree of each mapping population; thus, the progeny genotypes are expected, while the possible genotypes of germplasm accessions are not known a priori. Reproducibility was also comparable to that found in other studies employing the same array (99.8% for our control genotypes compared to 99–100% in Houel et al. 2015; Mercati et al. 2016; De Lorenzis et al. 2019). This very low error rate in the positive detection of SNPs is an evident advantage of this tool when compared to highly multiplexed, shallow sequencing strategies like GBS (Genotyping-By-Sequencing) and RAD (Restriction site-Associated DNA) sequencing that result in a high proportion of genotyping error, as well as missing data and under-calling of heterozygous sites (Mardis 2008; Barba et al. 2014; Hyma et al. 2015; Marrano et al. 2018).

Despite the successful genotyping rate and reproducibility, genotyping failure was observed for more than 1500 SNPs in each population (Table 1). The *Vitis*18K SNP array consists of approximately 25% loci identified from different *Vitis* species (Le Paslier et al. 2013), which could account for the low quality, or the lack of hybridization, for some SNP loci (De Lorenzis et al. 2015, 2019; Mercati et al. 2016; Sunseri et al. 2018). Accordingly, the fraction of non-*vinifera* SNPs loci among SNPs failing to genotype was greater than expected (52.9–54.7%), in agreement with the well-recognized low marker transferability in *Vitis* (Zou et al. 2020). Interestingly, a large proportion of SNPs failing to genotype were shared across the three mapping populations (Table S1). In conclusion, 1348 SNPs that failed in all three genotyped progenies are listed in this work (Table S1), which could support other researchers in SNP selection and deployment.

Concerning marker informativeness, we expected to find more informative markers in the DRxG1 mapping population that came from parents with higher genetic distance-a wine grape and a table grape selection. However, this cross had approximately 700 fewer informative markers than the CSxC and RRxCS populations, which is instead in agreement with a slightly lower heterozygosity of the two parents compared to the parents of the other two populations (‘Deckrot’ = 0.25, G1-7720 = 0.24, ‘Cabernet Sauvignon’ = 0.29, ‘Corvina’ = 0.31, ‘Rhine Riesling’ = 0.29). It is also possible that ascertainment bias during the SNP chip development reduced the number of informative markers in DRxG1. Most of the *Vitis vinifera* genotypes from which the SNP chip was developed are used for wine production, and only a few for table grapes. However, this would need to be investigated further.

### Mapping the SNPs of the *Vitis*18K SNP chip

Validation of SNP chromosomal location is required for the use of SNPs in marker-assisted selection as well as for genomics, trait mapping and gene cloning. Theoretical genomic positions can be deduced by BLASTN analysis of the SNP flanking regions. However, genetic mapping is required to validate positions. Theoretical positions on the latest 12X.v2 grapevine genome assembly were available in the literature for 10206 of the SNPs included in the *Vitis*18K SNP chip (Laucou et al. 2018). In this work, we integrated the BLASTN-derived positions on the ‘PN40024 12X.v2’ assembly for the additional 7859 SNPs (in agreement with those reported at https://urgi.versailles.inra.fr/jbrowse/; Table S1). Three genetic maps have been built so far using data derived from the *Vitis*18K SNP chip (Houel et al. 2015; Duchêne et al. 2020; Mamani et al. 2021). In these studies, 1122, 1587 and 1737 of the SNPs included in the *Vitis*18K SNP chip were mapped; however, the mapping data for only 1587 SNPs are available in tabular format. Thus, the largest part of the SNP markers in the *Vitis*18K array was not mapped previously. In the present study, we mapped 10890 SNPs. For most of these markers (86.6%), no mapping information had been reported. Most markers mapped showed a genetic position that was congruent to their expected theoretical position, which confirmed the SNP chromosomal assignment according to BLASTN of flanking sequences. We only identified a few SNPs which mapped to different chromosomes compared to their expected location (64 SNPs that are included in the 66 markers listed in Table S7) or which showed large inconsistencies (more than 10 cM) compared to the expected locations (27 SNPs, Table S8). Our SNP mapping results were highly congruent with the available published map data of Duchêne et al. (2020). Comparison of the ‘Riesling’ map from that work and ours resulted in a very high average Spearman rank correlation coefficient (0.9906) across linkage groups based on 784 common markers among which 764 were SNP markers genotyped by the *Vitis*18K SNP chip in both works (Table S14). Moreover, among the 66 markers mapping to chromosomes different from those expected that were identified in the present study (Table S7), two (ae_9_7573501 and ae_5_12606386) were also mapped in ‘Riesling’ by Duchêne et al. (2020) to the same alternative chromosome, further confirming our findings.

The scattered distribution across the genome of the SNPs mapping to chromosomes other than those established by BLASTN of flanking regions (Table S7a) suggests that their incorrect assignment might be related to short, repeated regions and consequent BLAST issues. Accordingly, our BLAST data show that more than one BLAST hit was found for 37 out of the 64 of the SNP flanking sequences. In more than half of these cases (21 out of 37), the alternative position established by genetic mapping was identified in the secondary BLAST hit(s) (in bold in Table S7b). Incorrect allocation by BLASTN mainly concerned SNP probes originating from species other than *Vitis vinifera*. Indeed, 71.9% of the SNPs that mapped to chromosomes different from those expected were identified in non-*vinifera* species, whereas non-*vinifera* SNPs represent only 25% in the *Vitis*18K SNP chip, again confirming low marker transferability across species within *Vitis* (Zou et al. 2020). Regarding markers with large intra-chromosomal inconsistencies compared to the expected position, such bias was lower, and we found two or more BLAST hits on the same chromosome in only three out of 27 cases (in bold in Table S8b), while we found clusters of linked SNPs in three different cases (Table S8a). Therefore, in general, the unexpected mapping positions can be attributed to less effective BLASTN searches due to cross-species markers as well as genome duplications. It is possible that these genome duplications are not present in the reference assembly, or present only in a specific parental genotype and associated mapping population. Conversely, inconsistent markers found in blocks of linked loci might indicate wrong alignment or orientation of scaffolds in the ‘PN40024 12X.v2’ assembly or translocations. In conclusion, in this work we were able to validate the theoretical genomic positions of 9431 SNPs not previously validated from the *Vitis*18K SNP chip by genetic mapping and provide a list of markers for which the theoretical physical position needs to be revised. This information is a valuable contribution to other genetic studies using the *Vitis*18K SNP chip. Finally, 281 markers assigned to the chromosome “chrUn” in the ‘PN40024 12X.v2’ genome were genetically mapped in this work to nine chromosomes, mainly to chromosomes 2, 7 and 10 (Table S9). Out of these, only 15 markers had been genetically positioned in previous studies (Houel et al. 2015; Duchêne et al. 2020), and these located on the same chromosomes that we identified. Similarly, most of the 76 GBS-derived variants physically assigned to the “Unknown” chromosome of the ‘PN40024 12X.v2’ genome that were genetically mapped by Tello et al. (2019), were placed in the same three chromosomes. The 281 markers were assigned to 104 different scaffolds stacked in the chromosome “ChrUn” (Table S9). Markers assigned to same scaffold were mapped in all cases to close genetic positions, and in several cases (193 markers) the same mapping was confirmed in more populations. Even though the current genome assembly (12X.v2) managed to assign 8% of the genome sequences unassigned in the previous assembly (12X.v0), 1692 scaffolds remained unanchored (Canaguier et al. 2017). The genetic position of the 281 originally unanchored markers mapped in the present work helps to anchor 104 of these scaffolds to the 19 chromosomes of the grapevine genome, with 86 scaffolds anchored in more than one population. This information can be used in building future versions of the grapevine reference genome.

### Developing an integrated map for grapevine based on the *Vitis*18K SNP chip

Despite the development of some genetic maps with data derived from the *Vitis*18K SNP chip already reported, no integrated map has been built so far to combine such information. To increase map marker density and provide a reliable shared marker order, we constructed an integrated reference map from three population maps. Two alternative approaches can be used in producing integrated genetic maps. The first, a “genetic merging approach”, makes use of recombination frequencies from genotypes to develop the integrated map in a similar fashion to bi-parental mapping, as is employed by the softwares Carthagene (De Givry et al. 2005), JoinMap (Van Ooijen 2009) and Lep-MAP3 (Rastas 2017). Alternatively, maps can be built from component maps by linear programming algorithms according to the “graph-method”, using marker positions and orders of the different population maps as starting information (Maccaferri et al. 2015), as is employed by the softwares MergeMap (Wu et al. 2008) and LPMerge (Endelman and Plomion 2014). In line with the previous reports (Tello et al. 2019), map integration starting from recombination frequencies, although providing a theoretical higher accuracy, proved too computationally demanding with large numbers of markers (> 5000) limiting the choice to the second approach. Therefore, to compensate for a lower integration accuracy of this approach, highly accurate population maps should be provided as well as a sufficient number of shared markers. A strategy incorporating a novel phasing system to reconstruct local haplotypes from adjacent bi-allelic SNPs to increase the percentage of fully informative multi-allelic markers to build accurate maps has been recently proposed for GBS-derived SNPs in grapevine (Tello et al. 2019). Here, even though an overall largely shared SNP ordering across the different maps with low number of conflicts was obtained using our strategy, some limits in building reliable shared orders at short distance, likely related to the low-informative nature of bi-allelic SNPs, were evident. On the other hand, in this study, the same markers were genotyped across the different subpopulations with the *Vitis*18K SNP chip. Focusing on the same marker set could represent an advantage, where other genotyping approaches like GBS, despite genotyping a much larger number of markers, do not necessarily obtain data for similar regions across individuals. Increasing the sequencing depth improves the shared marker density also in GBS genotyping, but requires suitable tools and approaches to ensure accuracy (Klápště et al. 2021).

The presented integrated map was constructed from a dataset including 6697 SNPs, of which 2713 markers were shared across at least two maps. For the map integration step, previous grapevine studies relied on the LPmerge software (Endelman and Plomion 2014), which aids linear conflict solving. Another “graph-method”-based software, MergeMap (Wu et al. 2008) has been widely used in other plant species despite typically estimating longer maps (Close et al. 2009; Galeano et al. 2011; Khan et al. 2012; Wang et al. 2014; Wen et al. 2017). In this study, we chose to apply this software, but we implemented a manually curated conflict solving step and included renaming of markers with different orders to avoid further map length inflation, which could result from order discrepancies (Maccaferri et al. 2015).

Using the described strategy, we produced an integrated map which included all 6697 SNPs with a total length of 2094.86 cM. The integrated map covers 98.8% of the grapevine genome, implying that most of distal ends of chromosomes are also represented. A significant lower coverage (93.8%) was only observed for chromosome 9 (Table 3, Fig. 5). Closer inspection confirmed that this reflected a lower marker density in the original population maps (Fig. 2). A similar under-representation of this region was also encountered in previous maps based on the *Vitis*18K SNP chip (Houel et al. 2015; Duchêne et al. 2020; Mamani et al. 2021). This is likely due to the fact that the majority of the markers at the distal end of chromosome 9 originate from the *Vitis* markers subset and were scored as monomorphic or failed in genotyping.

Our integrated map efficiently increased overall marker density compared to the three population maps including 3467, 3173 and 3459 representative SNP markers of the CSxC, DRxG1 and RRxCS populations, respectively. Compared to previous integrated maps available in grapevine, this map represents the densest integrated map for grapevine reported so far (Teh et al. 2017; Lewter et al. 2019; Tello et al. 2019; Zou et al. 2020). Moreover, additional genetic maps eventually developed based on the *Vitis*18K SNP chip can be easily further integrated based on the same approach described here. Despite the greater length we are reporting compared to previous integrated maps, this map provides a good uniform marker distribution across the genome (Fig. 5) and reduced gap lengths. Five to 9 gaps longer than 5 cM were observed in the population maps, due to long non-segregating stretches. Only one of the gaps, located on chromosome 13, was still retained in the integrated map, since this region was homozygous in both the CSxC and DRxG1 map. We can hypothesize that the applied genotyping strategy positively contributed to a homogeneous marker distribution and shorter gap length compared to other integrated maps (Teh et al. 2017; Lewter et al. 2019), since the SNP chip included SNPs pre-selected for a homogeneous genome coverage compared to random GBS-based genotyping (Le Paslier et al. 2013). We also explored the extent of LD by considering plants from all populations and observed this was largely reduced compared to that found in each population, indicating that a higher mapping resolution could be achieved by using the integrated map. However, the LD did not reach low values typically observed in accession collections in grapevine (Marrano et al. 2018). LD decayed below 0.2 on average within 600 kbp (ranging from 160 to 1360 Kbp across chromosomes, Figure S1), suggesting that the *Vitis*18K SNP chip would still provide a suitable tool for efficient trait mapping. Finally, released data can also support further simulation approaches relying on recombination, as well as, be used for testing and implementing also in grapevine LD-based strategies to position still unmapped SNP markers (Yadav et al. 2021).

A largely shared consensus order (uncolored regions in the final integrated map; Fig. 4, Table S10, Figure S2) was obtained, with only 88 markers across 31 genomic regions, highlighted in red, where no collinear marker order across maps could be deduced. The high consistency in marker order between the individual maps and integrated map demonstrates that the integrated map accurately represents the information from population maps. The integrated map showed a similar, even slightly higher, correlation to the assembly compared to that of the population maps. In order to provide information about the likelihood-of-fit of the integrated map, we also highlighted all markers potentially involved in generating conflicts to the assembly in the population maps. Blue regions include discrepancies with the assembly in original population maps. It is not possible to conclude whether discrepancies in such regions are due to inaccuracy in genetic data/assembly or to real genetic differences in any of the parents. Dark blue regions are those for which such discrepancies were shared across multiple maps, indicating a higher probability of inaccurately assembled short genomic portions (e.g., on chromosomes 4, 5, 8 and 13 where a few markers were incongruently allocated in all three genetic maps) or genetic differences shared in the different populations (e.g., on chromosome 3 and 5 in the CSxC and RRxCS populations, which share the ‘Cabernet Sauvignon’ parent). These areas should be investigated in more detail in future studies to establish the correct order in the respective genotypes.

In conclusion, in the present study, we document the genetic position of about 9500 markers included in the *Vitis*18K SNP chip tool and provide valuable information for the use of the *Vitis*18K SNP chip array. This is particularly valuable given the convenience and accuracy of high-throughput genotyping by cost-efficient SNP arrays. Furthermore, we release some interesting information for improving future grapevine genome assemblies. Finally, we explored the possibility to apply this tool as a standardized resource for breeding. By using three mapping populations, we constructed a reliable reference integrated SNP map, which represents the most saturated and high-density integrated genetic map thus far for cultivated grapevine. This integrated map will allow comparison of QTL locations of important phenotypic traits among different bi-parental populations, as well as fine mapping due to the increased marker density. Furthermore, it provides the foundation for establishing experiments of multi-parental QTL mapping as a valuable tool to increase power in QTL detection (Qu et al. 2021), contributing to a more comprehensive understanding of the genetic architecture of complex traits in this species.

## Supplementary Information

Below is the link to the electronic supplementary material.

Supplementary file 1. **Table S1**: List of SNP markers genotyped with the *Vitis*18K SNP chip. For each SNP marker the position on the ‘PN40024 12X.v0’ and ‘PN40024 12X.v2’ genome assembly is indicated with the corresponding source of information (columns D-H). The parental genotype (provided in TOP format as extracted from GenomeStudio), the outcome of the analysis performed in ASSiST, the segregation pattern, the outcome after the analysis performed in JoinMap® and the map position (as chromosome_cM) are reported for each marker in each population. For the dataset reduction, where markers with similar genotypes were excluded, the representative marker that was retained in each population is shown. The position in the integrated map is also indicated for each representative marker (as chromosome_cM). Finally, each marker is classified as congruent if it was mapped to the same chromosome of the theoretical position assigned to that marker within a genetic interval of ±10 cM. Abbreviations: chr = chromosome, CS = ‘Cabernet Sauvignon’, C = ‘Corvina’, DR = ‘Deckrot’, G1 = G1-7720, RR = ‘Rhine Riesling’, NA = not analyzed. **Table S2**: List of SSR markers genotyped in this study. For each marker, the reference and primer sequences and the position on the ‘PN40024 12X.v2’ genome assembly are indicated. The allele sizes (in bp) of the parents in each population, as well as the segregation pattern, the outcome after the analysis in JoinMap® and the map position for each marker are included. The position in the integrated map is also indicated (as chromosome_cM). Abbreviations: chr = chromosome, CS = ‘Cabernet Sauvignon’, C = ‘Corvina’, DR = ‘Deckrot’, G1 = G1-7720, RR = ‘Rhine Riesling’, NA = not analyzed. **Table S3**: Genotypic information (.loc file) of 142 progeny of the ‘Cabernet Sauvignon’ × ‘Corvina’ (CSxC) population for 7 464 informative markers (a), 137 progeny of the ‘Deckrot’ × G1-7720 (DRxG1) population for 6732 informative markers (b) and 139 progeny of the ‘Rhine Riesling’ × ‘Cabernet Sauvignon’ (RRxCS) population for 7461 informative markers (c). **Table S4**: Segregation patterns of the SNP and SSR markers in the three populations (CSxC: ‘Cabernet Sauvignon’ × ‘Corvina’, DRxG1: ‘Deckrot’ × G1-7720 and RRxCS: ‘Rhine Riesling’ × ‘Cabernet Sauvignon’), respectively. **Table S5**: Final population maps based on the whole dataset for CSxC (‘Cabernet Sauvignon’ × ‘Corvina’), DRxG1 (‘Deckrot’ × G1-7720) and RRxCS (‘Rhine Riesling’ × ‘Cabernet Sauvignon’). Abbreviation: chr = chromosome. The map position of each marker is indicated as chromosome_cM. **Table S6**: Evaluation of the population maps based on genetic data only (using the reduced dataset). Section (a) indicates the Spearman rank correlation coefficients between the population maps (obtained from genetic data only) and the ‘PN40024 12X.v2’ genome assembly. Section (b) refers to chromosome 8 from DRxG1 parental maps (P1 = ‘Deckrot’, P2 = G1-7720) before and after the integration of assembly information (fixed order). The table reports some map quality parameters (map length in cM, maximum NN Fit/Stress values in cM) and Spearman rank correlation coefficients between the maps and the ‘PN40024 12X.v2’ assembly. In both sections, maps with poor correlation to the ‘PN40024 12X.v2’ assembly are indicated in bold. Abbreviations: chr = chromosome, CSxC = ‘Cabernet Sauvignon’ × ‘Corvina’, DRxG1 = ‘Deckrot’ × G1-7720 and RRxCS = ‘Rhine Riesling’ × ‘Cabernet Sauvignon’. **Table S7**: Comprehensive list of markers mapping to a different chromosome from that previously reported on the ‘PN40024 12X.v2’ genome assembly. Section a) indicates for each marker the expected physical position as well as the genetic position in the CSxC (‘Cabernet Sauvignon’ × ‘Corvina’), DRxG1 (‘Deckrot’ × G1-7720) and RRxCS (‘Rhine Riesling’ × ‘Cabernet Sauvignon’) maps, respectively. Markers where the alternative chromosome assignment is confirmed by mapping in two or more populations are shown in bold. Section (b) indicates the highest BLASTN result together with alternative BLASTN results. Secondary BLASTN hits supporting the alternative chromosome genetic assignments are indicated in bold. Abbreviations: chr = chromosome, pos = position (in bp in column C, in cM in columns E, G, I of section A). **Table S8**: Markers with inconsistencies larger than 10 cM in comparison with the reference genome assembly. Section a) lists the markers with the mapped position in the CSxC (‘Cabernet Sauvignon’ × ‘Corvina’), DRxG1 (‘Deckrot’ × G1-7720) and RRxCS (‘Rhine Riesling’ × ‘Cabernet Sauvignon’) maps, respectively. Marker inconsistencies, which were confirmed in more than one population, are indicated in bold. Section b) lists the expected physical position (sstart-send) of these markers on chromosomes (sseqid), according to BLASTN results. Bold indicates multiple hits in the same chromosome where the marker was mapped. Abbreviations: chr = chromosome, pos = position (in bp in column C, in cM in columns E, G and I of section A). **Table S9**: Markers physically assigned to the “ChrUn” grapevine chromosome (columns B-C) and now genetically assigned to chromosomes from 1 to 19 in one or more populations (columns D-I). Column J reports the anchoring of each marker to one of the 104 scaffolds located to the Unknown chromosome from the Vitisagp grapevine genome assembly (https://urgi.versailles.inra.fr/Species/Vitis/Data-Sequences/Genome-sequences, Canaguier et al. 2017). Abbreviations: chr = chromosome, pos = position (in bp in column C, in cM in columns E, G and I), CSxC = ‘Cabernet Sauvignon’ × ‘Corvina’, DRxG1 = ‘Deckrot’ × G1-7720 and RRxCS = ‘Rhine Riesling’ × ‘Cabernet Sauvignon’. **Table S10**: List of markers that were non-collinear between population maps. The table reports for each marker the original name, the name with the suffix used in the integrated map (including the information on one or both the conflict(s), in letters as explained in Materials and Methods, and the number of population maps where it was mapped, within brackets), the physical position in the ‘PN40024 12X.v2’ genome assembly, the genetic position in the maps where it was mapped, the two genetic positions in the integrated map and the conflict type. Abbreviations: chr = chromosome, pos = position (in bp in column E, in cM in columns G, I, K, M and N), CSxC = ‘Cabernet Sauvignon’ × ‘Corvina’, DRxG1 = ‘Deckrot’ × G1-7720 and RRxCS = ‘Rhine Riesling’ × ‘Cabernet Sauvignon’. **Table S11**: List of markers whose genetic position was in conflict with the assembly in at least one of the population maps, which were based on the reduced datasets of markers and were used to construct the integrated map. The table reports for each marker the original name, the name with the suffix used in the integrated map (including the information on one or both the conflict(s) type, in letters as explained in Materials and Methods, and the number of population maps where it was mapped, within brackets), the physical position in the ‘PN40024 12X.v2’ genome assembly, the number of population maps where it was mapped, the map where the conflict(s) occurred and the genetic position on the integrated map (indicated as chromosome_cM). Markers involved in conflicts to the assembly for which the conflict was originally revealed in more than one map (not introduced by the conflict solving step) are shown in dark blue, while conflictual markers affecting just one specific map, or the shared ones introduced by forcing marker orders during conflict solving are shown in light blue. Markers for which the support for the alternative marker order was introduced in more maps by forcing the orders during the conflict solving step and for which alternative orders are likely were not labeled. Abbreviations: chr = chromosome, pos = position (in bp in column D, in cM in column I). **Table S12**: Grapevine integrated map based on three mapping populations (CSxC: ‘Cabernet Sauvignon’ × ‘Corvina’, DRxG1: ‘Deckrot’ × G1-7720 and RRxCS: ‘’Rhine Riesling’ × ‘Cabernet Sauvignon’). Markers in the population genetic map(s) that are mapped in conflictual positions compared to their physical positions are represented in blue (dark blue if supported by more than one population map, shared conflicts introduced by conflict solving were not highlighted), while markers involved in conflicts across maps are coded in red. Priority is given to red labeling. Abbreviation: chr = chromosome. Numbers within brackets indicate the number of population maps in which the markers are mapped. The position of each marker in the integrated map is indicated as chromosome_cM. **Table S13**: Spearman rank correlation coefficients between the population maps and the integrated map. Abbreviations: chr = chromosome, CSxC = ‘Cabernet Sauvignon’ × ‘Corvina’, DRxG1 = ‘Deckrot’ × G1-7720 and RRxCS =‘Rhine Riesling’ × ‘Cabernet Sauvignon’. **Table S14**: Comparison of ‘Riesling’ maps obtained in Duchêne et al. (2020) and in this study. Section (a) reports chromosome assignment and map position (in cM) for 784 common markers. Markers mapping in both studies to the same alternative chromosome compared to that expected are shown in bold. Section (b) indicates the Spearman rank correlation coefficients between the two maps calculated separately for each chromosome and as an average value across chromosomes. Abbreviations: chr = chromosome.

Supplementary file 2. **Figure S1**: Decay of LD estimated for each of the 19 grapevine chromosomes. A regression line for the average r2 value estimated in sequential bins of 20 Kb against the physical distances between SNP is shown for each of the three populations. Decay in CSxC (‘Cabernet Sauvignon’ × ‘Corvina’, 142 plants) is shown in blue, in DRxG1 (‘Deckrot’ × G1-7720, 137 plants) in red and in RRxCS (‘Rhine Riesling’ × ‘Cabernet Sauvignon’, 139 plants) in green, respectively. The LD decay for the population built including individuals from each of the three populations (418 plants) is shown in yellow. Abbreviations: chr = chromosome. **Figure S2**: Detailed grapevine integrated map based on three mapping populations (CSxC: ‘Cabernet Sauvignon’ × ‘Corvina’, DRxG1: ‘Deckrot’ × G1-7720 and RRxCS: ‘Rhine Riesling’ × ‘Cabernet Sauvignon’) indicating the names of all non-collinear (with the ‘PN40024 12X.v2’ assembly and between populations) markers and markers mapped in all three populations (bold). Markers that are in non-collinear position with the 'PN40024 12X.v2’ genome assembly order are indicated in blue (dark blue if supported by more than one population map, shared conflicts introduced by conflict solving are not highlighted). The population map involved in the conflict is indicated with suffix –A (CSxC), –B (DRxG1) or –C (RRxCS). Markers that are in non-collinear positions between population maps are indicated in red with the suffixes –AB (CSxC vs DRxG1), –AC (CSxC vs RRxCS) or –BC (DRxG1 vs RRxCS) to indicate conflictual populations. Priority is given to red labeling. The suffixes (1) (2) (3) indicate the number of population maps in which the markers were mapped. Markers that are collinear with the ‘PN40024 12X.v2’ assembly and between population maps and are not mapped in all three populations are only indicated with a line.

## Data Availability

The genotyping data (.loc file) are available as supplementary information.
