# Supplementary material for: A high-density integrated map for grapevine based on three mapping populations genotyped by the Vitis18K SNP chip
Source: Theor Appl Genet. 2022 Oct 21;135(12):4371–90. doi: 10.1007/s00122-022-04225-6 (PMC9734222; doi:10.1007/s00122-022-04225-6)
Supplement: Supplementary file 2 — Figure S1: Decay of LD estimated for each of the 19 grapevine chromosomes. A regression line for the average r2 value estimated in sequential bins of 20 Kb against the physical distances between SNP is shown for each of the three populations. Decay in CSxC (‘Cabernet Sauvignon’ × ‘Corvina’, 142 plants) is shown in blue, in DRxG1 (‘Deckrot’ × G1-7720, 137 plants) in red and in RRxCS (‘Rhine Riesling’ × ‘Cabernet Sauvignon’, 139 plants) in green, respectively. The LD decay for the population built including individuals from each of the three populations (418 plants) is shown in yellow. Abbreviations: chr = chromosome. Figure S2: Detailed grapevine integrated map based on three mapping populations (CSxC: ‘Cabernet Sauvignon’ × ‘Corvina’, DRxG1: ‘Deckrot’ × G1-7720 and RRxCS: ‘Rhine Riesling’ × ‘Cabernet Sauvignon’) indicating the names of all non-collinear (with the ‘PN40024 12X.v2’ assembly and between populations) markers and markers mapped in all three populations (bold). Markers that are in non-collinear position with the 'PN40024 12X.v2’ genome assembly order are indicated in blue (dark blue if supported by more than one population map, shared conflicts introduced by conflict solving are not highlighted). The population map involved in the conflict is indicated with suffix –A (CSxC), –B (DRxG1) or –C (RRxCS). Markers that are in non-collinear positions between population maps are indicated in red with the suffixes –AB (CSxC vs DRxG1), –AC (CSxC vs RRxCS) or –BC (DRxG1 vs RRxCS) to indicate conflictual populations. Priority is given to red labeling. The suffixes (1) (2) (3) indicate the number of population maps in which the markers were mapped. Markers that are collinear with the ‘PN40024 12X.v2’ assembly and between population maps and are not mapped in all three populations are only indicated with a line. [file 122_2022_4225_MOESM2_ESM.pdf]

Supplementary data

A high-density integrated map for grapevine based on three mapping populations genotyped by the *Vitis*18K SNP chip

Jessica A Vervalle[1,2*], Laura Costantini[3*], Silvia Lorenzi[3], Massimo Pindo[3], Riccardo Mora[4], Giada Bolognesi[4], Martina Marini[4], Justin G Lashbrooke[5], Ken R Tobutt[2], Melané A Vivier[5], Rouvay Roodt-Wilding[1], Maria Stella Grando[3,6], Diana Bellin[4ǂ]

[1] Department of Genetics, Stellenbosch University, Stellenbosch 7600, South Africa

[2] ARC Infruitec-Nietvoorbij, Stellenbosch 7599, South Africa

[3] Research and Innovation Centre, Fondazione Edmund Mach, San Michele all'Adige, Italy

[4] Department of Biotechnology, University of Verona, Verona, Italy

[5] South African Grape and Wine Research Institute, Stellenbosch University, Stellenbosch 7600, South Africa

[6] Center Agriculture Food and Environment (C3A), University of Trento, San Michele all'Adige, Italy

[*]Jessica A Vervalle and Laura Costantini contributed equally to this paper

[ǂ]Corresponding author: diana.bellin@univr.it

Figure S1: Decay of LD estimated for each of the 19 grapevine chromosomes. A regression line for the average r^2 value estimated in sequential bins of 20 Kb against the physical distances between SNP is shown for each of the three populations. Decay in CSxC ('Cabernet Sauvignon' x 'Corvina', 142 plants) is shown in blue, in DRxG1 ('Deckrot' x G1-7720, 137 plants) in red and in RRxCS ('Rhine Riesling' x 'Cabernet Sauvignon', 139 plants) in green, respectively. The LD decay for the population built including individuals from each of the three populations (418 plants) is shown in yellow. Abbreviations: chr = chromosome.

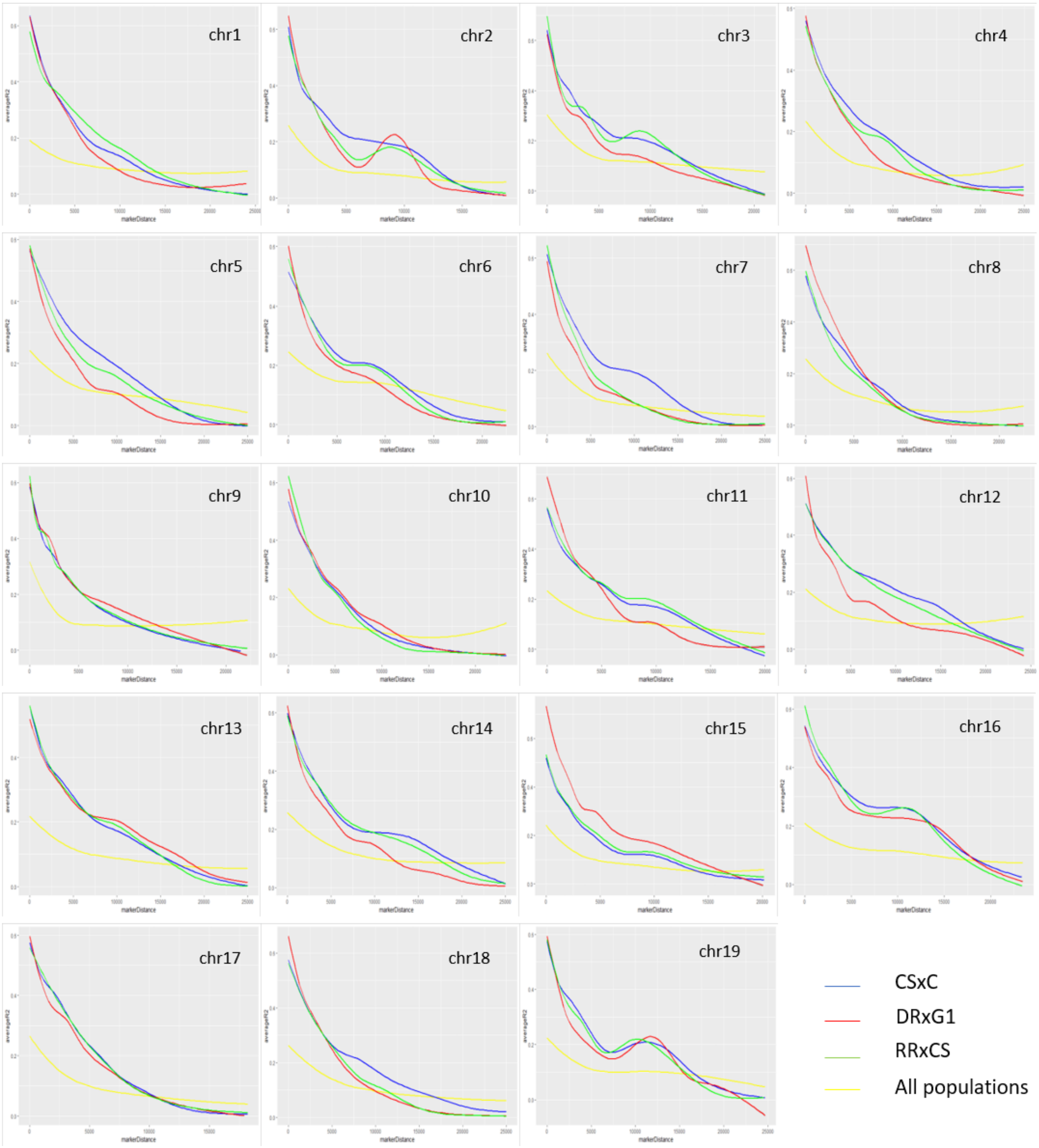

Figure S2: Detailed grapevine integrated map based on three mapping populations (CSxC: 'Cabernet Sauvignon' x 'Corvina', DRxG1: 'Deckrot' x G1-7720 and RRxCS: 'Rhine Riesling' x 'Cabernet Sauvignon') indicating the names of all non-collinear (with the 'PN40024 12X.v2' assembly and between populations) markers and markers mapped in all three populations (bold). Markers that are in non-collinear positions with the 'PN40024 12X.v2' genome assembly order are indicated in blue (dark blue if supported by more than one population map, shared conflicts introduced by conflict solving are not highlighted). The population map involved in the conflict is indicated with suffix –A (CSxC), –B (DRxG1) or –C (RRxCS). Markers that are in non-collinear positions between population maps are indicated in red with the suffixes –AB (CSxC vs DRxG1), –AC (CSxC vs RRxCS) or –BC (DRxG1 vs RRxCS) to indicate conflictual populations. Priority is given to red labeling. The suffixes (1) (2) (3) indicate the number of population maps in which the markers were mapped. Markers that are collinear with the 'PN40024 12X.v2' assembly and between population maps and are not mapped in all three populations are only indicated with a line.

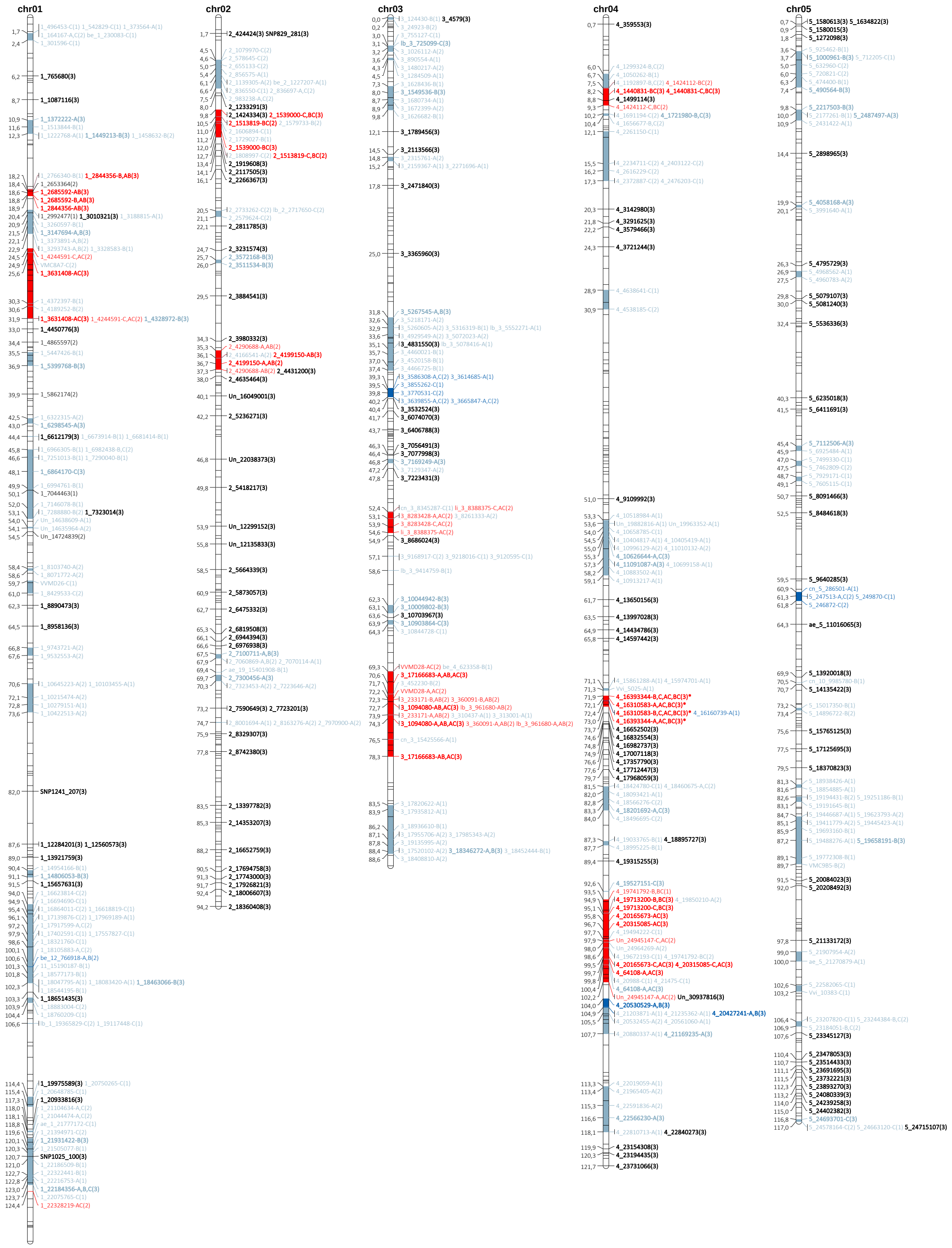
chr01
chr02
chr03
chr04
chr05

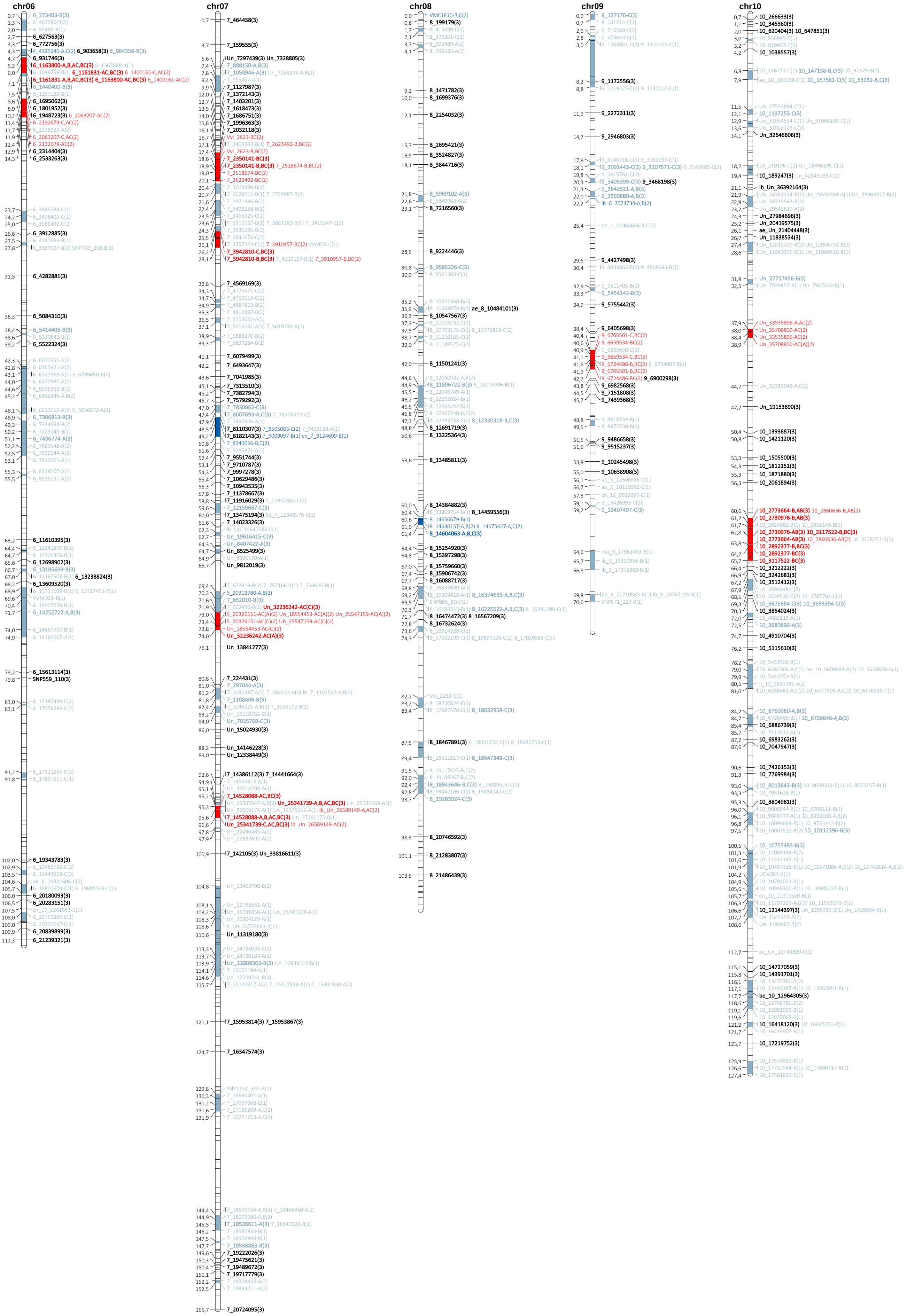
chr06
chr07
chr08
chr09
chr10

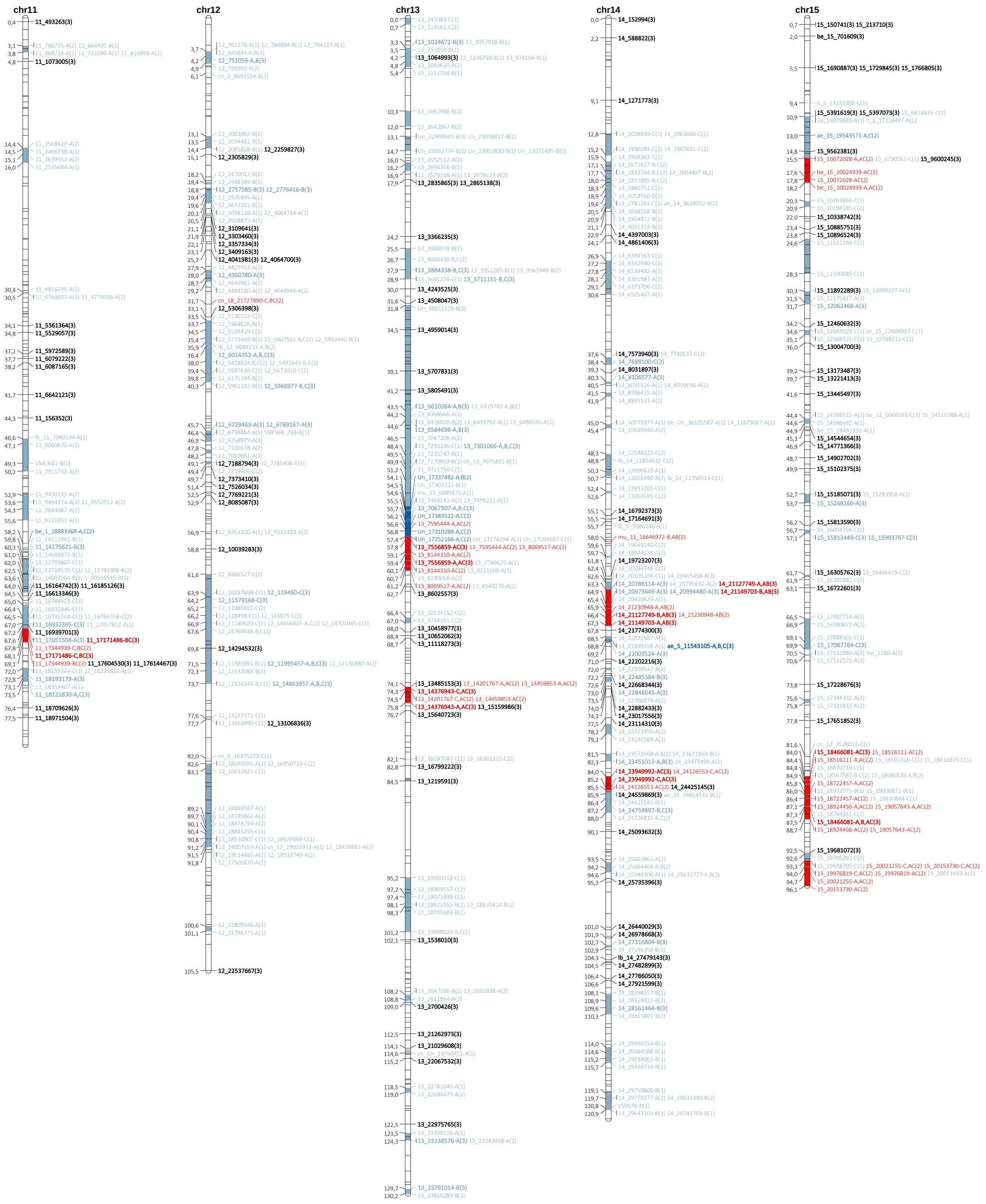
chr11
chr12
chr13
chr14
chr15

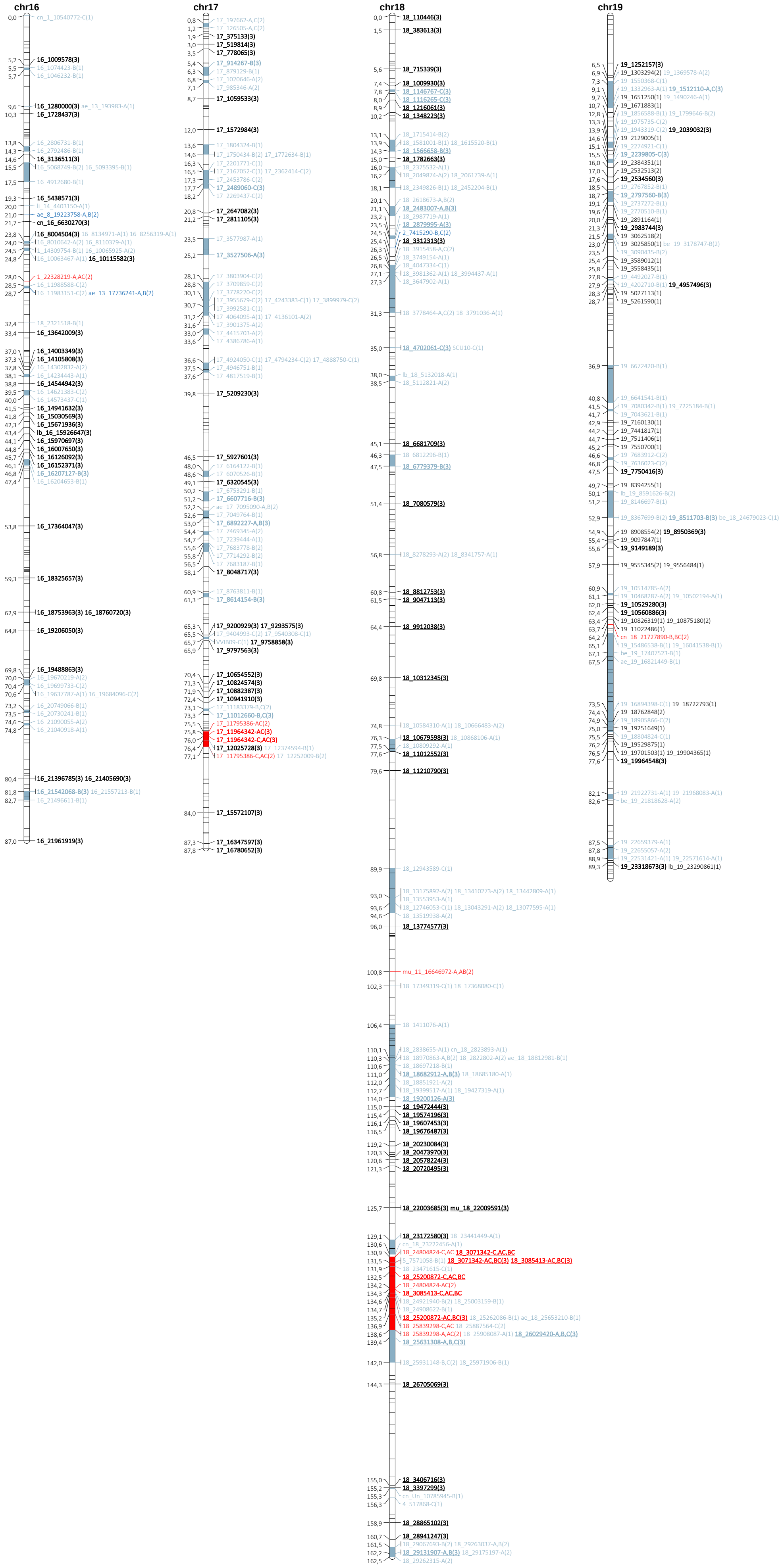
chr16
chr17
chr18
chr19
